# SLC43A2 and NFκB signaling pathway regulate methionine/cystine restriction-induced ferroptosis in esophageal squamous cell carcinoma via a feedback loop

**DOI:** 10.1038/s41419-023-05860-7

**Published:** 2023-06-03

**Authors:** Hao Peng, Yuyu Yan, Min He, Jinxia Li, Lianghai Wang, Wei Jia, Lan Yang, Jinfang Jiang, Yunzhao Chen, Feng Li, Xianglin Yuan, Lijuan Pang

**Affiliations:** 1 Department of Oncology, Tongji Hospital, Tongji Medical College, Huazhong University of Science and Technology, 430030 Wuhan, Hubei Province China; 2 NHC Key Laboratory of Prevention and Treatment of Central Asia High Incidence Diseases (First Affiliated Hospital, School of Medicine, Shihezi University)/Department of Pathology and Key Laboratory for Xinjiang Endemic and Ethnic Diseases, Shihezi University School of Medicine, 832002 Shihezi, China; 3 The People’s Hospital of Suzhou National Hi-Tech District, 215010 Suzhou, China; 4 Department of Pathology and Medical Research Center, Beijing Chaoyang Hospital, Capital Medical University, 100020 Beijing, China; 5 Department of Pathology, Central People’s Hospital of Zhanjiang and Zhanjiang Central Hospital, Guangdong Medical University, 524000 Zhanjiang, Guangdong China

**Keywords:** Cancer metabolism, Prognostic markers

## Abstract

Studies have indicated dietary restriction of methionine/cystine provided a therapeutic benefit in diseases such as cancer. However, the molecular and cellular mechanisms that underlie the interaction between methionine/cystine restriction (MCR) and effects on esophageal squamous cell carcinoma (ESCC) have remained elusive. Here, we discovered the dietary restriction of methionine/cystine has a large effect on cellular methionine metabolism as assayed in a ECA109 derived xenograft model. RNA-seq and enrichment analysis suggested the blocked tumor progression was affected by ferroptosis, together with the NFκB signaling pathway activation in ESCC. Consistently, GSH content and GPX4 expression were downregulated by MCR both in vivo and in vitro. The contents of Fe^2+^ and MDA were negatively correlated with supplementary methionine in a dose-dependent way. Mechanistically, MCR and silent of SLC43A2, a methionine transporter, diminished phosphorylation of IKKα/β and p65. Blocked NFκB signaling pathway further decreased the expression of SLC43A2 and GPX4 in both mRNA and protein level, which in turn downregulated the methionine intake and stimulated ferroptosis, respectively. ESCC progression was inhibited by enhanced ferroptosis and apoptosis and impaired cell proliferation. In this study, we proposed a novel feedback regulation mechanism underlie the correlation between dietary restriction of methionine/cystine and ESCC progression. MCR blocked cancer progression via stimulating ferroptosis through the positive feedback loop between SLC43A2 and NFκB signaling pathways. Our results provided the theoretical basis and new targets for ferroptosis-based clinical antitumor treatments for ESCC patients.

## Introduction

Globally, China has the highest incidence and mortality rate of esophageal cancer. More than 90% of the cases were histologically classified as esophageal squamous cell carcinoma (ESCC) [1, 2]. Despite there were advances in the early diagnosis and clinical treatment of ESCC recently, the 5-year survival rate of patients with esophageal cancer was still less than 20% [3]. It’s urgent to explore the molecular mechanisms and potential therapeutic targets to improve the effects of ESCC treatment. In recent years, more and more therapeutic strategies had focused on an important feature of cancer—tumor metabolic reprogramming [4]. Tumor cells grow rapidly, so it needed to synthesize large amounts of biological macromolecules and energies in the process of their progress. Therefore, various anabolisms process involving amino acids played an important role during tumor development [5]. As the key regulatory mechanisms affecting tumor amino acid metabolism were numerous and complex, it had not yet been fully elucidated.

There were growing evidence that differences in the utilization of dietary nutrients, such as amino acids, have a profound impact on tumor metabolism, growth, and treatment outcomes [6–11]. Sulfur-containing amino acids (SAAs) include methionine, cysteine, homocysteine, and taurine. It has been shown that culture conditions that restrict sulfur-containing amino acids made K-RAS-transformed cells more susceptible to oxidative stress [12]; and a high sulfur diet increases the risk of colorectal cancer [13]. Methionine and cystine are the only amino acids involved in protein synthesis. Methionine was the first amino acid used in eukaryotic protein synthesis, while cysteine contributes to the formation of tertiary or quaternary protein structures by forming disulfide bonds with other cysteine residues. As an essential amino acid, methionine must be consumed through the diet [14, 15], whereas cystine is a semi-essential amino acid that can be obtained from diet or by de novo synthesis from methionine and serine via the transsulfuration pathway, with cystine serving primarily as a precursor to glutathione [16]. Glutathione, a reducing molecule synthesized from glycine, cysteine, and glutamate, played a key role in the defense against reactive oxygen species, nutrient deprivation and heavy metal stress [11]. Therefore, it is crucial to investigate the specific mechanisms and functions of SAAs, especially methionine metabolism, affecting cancer.

Recent studies have shown that variety types of cancers could be prevented by restricting dietary methionine or cystine, two sulfur-containing essential amino acids [17–22]. This dietary intervention affected the metabolic levels in one-carbon metabolism in tumor cells and adversely affect tumor progression, ultimately sensitizing certain human cancer cells to chemotherapy and radiotherapy by means of tumor cell autoregulation [23]. Oxidation–reduction homeostasis of Ras-transformed mouse fibroblasts (NIH3T3-RAS cells) was disrupted and they were unable to grow normally in the absence of methionine or cystine, which further suggests that the enhanced oncogenic potential of cells depended on the involvement of methionine or cystine [12]. However, there were varying degrees of methionine/cystine dependence in human cancer cells [24], and we were still unsure of the underlying molecular mechanisms.

The dietary interventions could be used as one of the therapeutic tools to treat various types of diseases, from yeast to mammals [25–27]. The reduction of methionine or cystine intake protects non-tumor cells and restores the immune surveillance system against cancer [28]. In breast cancer, oncogenic PIK3CA contributed to increased methionine dependence in tumor cells through inhibition of xCT (a cystine transporter protein) expression [29]. A study further demonstrated that cancer methionine restriction regulated T-cell antitumor activities by increased STAT5 expression through restored H3K79me2 [30]. These findings provided strong evidences that dietary interventions may prevent cancer via regulating the expression of various cancer-related genes. Phase I clinical trials have assessed the safety and tolerability of methionine restriction as a personalized nutritional approach to treating cancer [31]. The exploration of methionine/cystine-restricted diet in the treatment of ESCC was still in the primary stage, the mechanisms of methionine and cystine in regulating ESCC progression needed to be further studied.

Here, we observed that methionine/cystine deprivation inhibited ESCC progression and facilitated ferroptosis through a positive feedback loop between SLC43A2 and NFκB signaling pathway in ESCC. A methionine/cystine-deprived diet remarkable inhibitory effects were observed in methionine metabolism activities and tumor proliferation in MCR (methionine and cystine restriction) group when compared with CTR (control diet) group in a ECA109 xenografts model. Results showed methionine/cystine deprivation may affect tumor progression by regulating NFκB signaling pathway and cellular ferroptosis in vivo and in vitro. Mechnistically, diminished methionine metabolism activities and decreased the expression of p-IKKα/β and p-p65 in ESCC. The blocked NFκB signaling pathway further restrained the expression of SLC43A2 and GPX4. Collectively, our findings suggested that methionine/cystine dietary intervention blocked the proliferation of ESCC may through inducing ferroptosis via a positive feedback loop between SLC43A2 and NFκB signaling pathway, which could be a potential therapeutic approach against cancer progression and provided a theoretical basis for individualized treatment of ESCC patients.

## Materials and methods

### Reagents and antibodies

The following antibodies were used: SLC43A2 (Novusbio-NBPI-82706, CO, USA); p65 (Ab32536), phospho-p65 (Ab86299), IKKα/β (Ab32041), GPX4 (Ab125066), SLC7A11 (Ab175186), Bax (Ab32503) and cleaved PARP (Ab32561) were from Abcam (Cambridge, UK); phospho-IKKα/β (Ser176/180) (2697) was from Cell Signaling Technology (MA, USA); Bcl-2 (AB112) was from Beyotime (China); β-actin (RG000120) was from Solarbio (China).

Enzyme-linked immunosorbent assay (ELISA) kits (MEIMIAN, Jiangsu, China) for Glutathione (MM-0661M1), Methionine (MM-45354M1), S-adenosylhomocysteine (MM-43942M1), S-adenosylmethionine (MM-1072M1) and Homocysteine (MM-0898H2) were used to detect the corresponding substances in mouse blood and tumor tissues or ESCC cells. CCK-8 (Solarbio, Beijing, China) was used to detect the proliferation of cells. Malondialdehyde (MDA) assay kit (TBA method) (A003-1), glutathione (GSH) assay kit (A006-2-1) and the iron assay kit (A039-2-1) were purchased from Nanjing Jiancheng (Jiangsu, China) and used for detection of cellular ferroptosis. Bay 11-7082 was purchased from Merck (NJ, USA). Ferrostatin-1 was purchased from Sigma (NJ, USA).

### Cell culture and transfection

ECA109 and EC9706, human ESCC cell lines, were obtained from the Chinese Academy of Sciences (Shanghai, China). The cells were examined for authenticity by STR. Cells were cultured in DMEM (11965, Thermo Fisher, MA, USA) supplemented with 10% FBS or DPBS, penicillin (100 U/mL), and streptomycin (100 mg/mL) at 37 °C under 5% CO_2_. Prior to experiments, all cell lines were obtained more than 1 year in advance and propagated for fewer than 6 months after being thawed. In methionine/cystine restriction assay, DMEM with no glutamine, no methionine, and no cystine (21013, Thermo Fisher, MA, USA) were used, 10% FBS, 2 mM l-glutamine (Solarbio, Beijing, China) and methionine (10 μM, 20 μM, 30 μM, 50 μM or 100 μM) (Solarbio, Beijing, China) were added according to experimental design. In order to prevent the dead cells from gathering into clumps and misleading the results, attentions needed to paid to the following points during the experiment: the cell growth density should not exceed 80%; operated gently to prevent the cells from falling off in pieces; if clumps of cells were visible in the cell suspension, left it for about half a minute and aspirated the upper part of the suspension without clumps of cells for subsequent manipulations. These operation points were also applicable to other experiments in this study.

The SLC43A2 siRNA (sc-94194, SANTA CRUZ, TX, USA) was transfected into ECA109 and EC9706 cells and incubated in complete medium or MCR medium (same formula as complete medium except for methionine (10 μM) and cystine (0 μM)) according to the experimental requirements.

### Xenograft model for testing efficacy of methionine/cystine restriction

All animal experimental protocols were approved by the animal experimental ethical inspection of the first affiliated hospital, Shihezi University School of Medicine (A2022-033-01). The SPF-grade male BALB/c nude mice (4–6 weeks) were provided by Guangdong Animal Experiment Center. In accordance with the GB14925-2010 national standard (China), mice were fed in an enclosed environment, the temperature (23 ± 2 °C) and humidity (60 ± 5%) were constant, and a 12 h light/dark cycle was followed. After the mice completed their acclimatization, they were randomly divided into two groups of five mice each, into a methionine/cystine restriction (MCR) group and a control diet (CTR) group. Mice were fed with specific food during the experiment, the MCR group was fed a methionine (0.12%) and cystine (0%) diet (TP0033; Trophic Animal Feed High-tech Co., Ltd. China); whereas the CTR group was fed a methionine- and cystine-supplement diet (TP0032S; Trophic Animal Feed High-tech Co., Ltd. China). During the study period, food and water were provided ad libitum.

ECA109 cell line was injected subcutaneously in the axilla of nude mice at 200 μL/each (to ensure that each mouse was inoculated with 2 million cells); when the axillary tumor diameter reached about 0.1–0.2 cm (until the requirement was reached, the nude mice were fed uniformly with ordinary feed), the nude mice were divided into two groups in random: one group for the MCR group and one group for the CTR group, while the feed was changed. On day 14 and day 31 after cell line injection, respectively, nude mice from each of the two groups were randomly selected and anesthetized for in vivo imaging to observe the growth of in situ transplanted tumors. Note: GFP signal expression was observed with the IVIS-Lumina-Series-III Live Imaging System. After placing the anesthetized mice into the imaging platform, the illumination is turned off and the specific photons emitted in the nude mice are photographed in the dark field. They were sterilized and put back after the completion of the imaging operation. The mice were closely observed for general status, mobility, hair quantity and luster, diet and drinking water, etc. The mice in different treatment groups were weighed and monitored for subcutaneous implantation tumor volume every 3 days, and uniform records were made. The indicators of experimental discontinuation were 31 days after tumor cell injection, or a certain tumor reached 1500 mm^3^, or 3 or more mice in the experimental group died. At the end of the experiment, the animals were euthanized by cervical dislocation.

### RNA-sequencing (RNA-Seq) library preparation and data analysis

For RNA-Seq library preparation, total RNA was extracted as previously described from MCR and CTR tissues (*n* = 3 each) [32]. After extracting total RNA and digesting DNA with DNase, mRNA was enriched with magnetic beads with Oligo (dT), mRNA was broken into short fragments by adding interruption reagent, and the interrupted mRNA was used as a template to synthesize one-stranded cDNA with six-base random primers, then a two-stranded cDNA was synthesized by preparing a two-stranded synthesis reaction system, and double-stranded cDNA was purified by using a kit. The purified double-stranded cDNA was then end-repaired, a-tailed and connected to the sequencing junction, followed by fragment size selection and finally PCR amplification; the constructed library was quality-checked with an Agilent2100 Bioanalyzer and sequenced using an Illumina HiSeqTM2500 sequencer.

Cufflinks software was used to identify transcript assembly and differentially expressed genes [33]. The CummeRbund package in R was used to analyze the RNA-Seq data [34]. The DAVID Functional Annotation Clustering Tool was used to analyze the functional enrichment of differentially expressed genes. Heatmap Builder was used to generate heat maps of differentially expressed genes [35].

### ELISA assay in tumor tissue and plasma

Tumor tissue pretreatment: take an appropriate amount of tissue block, wash it in pre-cooled PBS (0.02 mol/L, pH 7.0–7.2) to remove blood, and weigh it for later use (tissue block). A variety of homogenization methods can be used at the same time to achieve better crushing effect: first, move the tissue block into a glass homogenizer, add 5–10 mL of pre-cooled PBS (tissue and the mass-to-volume ratio of PBS is recommended to be 1:5, that is, 1 g of sample is added to 5 mL of PBS) for sufficient grinding, and this process needs to be carried out on ice. Centrifuge the prepared homogenate at 5000×*g* for 5 min, and collect the supernatant for detection. Test the tumor tissue supernatant and mouse serum obtained by the treatment according to the kit instructions.

### Western blot analysis

Western blot was operated as previously described [36]. Homogenized tissues or cells were lysed at 4 °C in radioimmunoprecipitation assay buffer (Solarbio, R0010), and protease and phosphatase inhibitors were added. Protein lysates were resolved by SDS–PAGE and transferred to Immun-Blot PVDF membranes (Solarbio). Membranes were blocked in buffer (5% nonfat milk) for 1 h. Then overnight with certain primary antibodies at 4 °C. Followed by incubation with secondary antibody (Solarbio).

The primary antibodies were: SLC43A2 (1:2000), p65 (1:10000), Phospho-p65 (1:5000), IKKα/β (1:5000), IKKα/β (Ser176/180) (1:1000), GPX4 (1:250), SLC7A11 (1:2000), Bax (1:1000), cleaved PARP (1:10000), Bcl-2 (1:1000), and β-actin (1:1000).

### Quantitative real-time polymerase chain reaction (qRT-PCR)

RNA was extracted from cells using DNA Extraction Kit (Qiagen) following the manufacturer’s instructions. The primers were as followed: SLC43A2: 5’-TGCACCGCTGTGTTGGAAA-3’ and 5’-CCGTGCTGTTAGTGACATTCTC-3’; GPX4: 5′-CTGCTCTGTGGGGCTCTG-3′ and 5′-ATGTCCTTGGCGGAAAACTC-3′; ACSL1: 5′-CTTATGGGCTTCGGAGCTTTT-3′ and 5′-CAAGTAGTGCGGATCTTCGTG-3′; ACSL5: 5’-GCTTATGAGCCCACTCCTGATG-3’ and 5’-GGAAGAATCCAACTCTGGCTCC-3’; GAPDH: 5′-ACAGTCAGCCGCATCTTCTT-3′ and 5′-AATTTGCCATGGGTGGAAT-3′. cDNA quantitative RT-PCR kit and SYBR Green master mix (Takara, Tokyo, Japan) were used. The cycle threshold (Ct) values were normalized to expression levels of GAPDH.

### Colony formation assay

For colony formation assay, after transfection, 1000 viable cells were plated in six-well plates in triplicate and maintained in a complete medium for 15 days. Foci were fixed with 4% polyoxymethylene (Solarbio, P8430) and stained with 0.1% crystal violet (Solarbio, G1061).

### CCK-8 assay

For CCK-8 assay, 4000 ESCC cells were seeded for each well of a 96-well plate. According to the experimental design, after cells cultured in medium contained 10 μM, 20 μM, 30 μM, 50 μM or 100 μM methionine for different time periods, 10 µl of CCK-8 agents were added to each well, and cells were incubated for 2 h without any light and optical density was measured at a wavelength of 450 nm.

### Detection of cellular ferroptosis-related molecules

ECA109 and EC9706 cells were cultured for 72 h. The detections of Fe^2+^, MDA, and GSH were carried out in accordance with the product manuals. Briefly, the cells were broken using PBS as homogenization medium and then centrifuged, and the supernatant was taken for the assay. After adding the corresponding reagents according to the instructions, mixing and centrifugation, the experiment was performed using a spectrophotometer. The experimental grouping is described in figure legends.

### Terminal deoxynucleotidyl transferase dUTP nick end labeling (TUNEL) assay

TUNEL assay was performed on ESCC cells. Apoptotic cells were detected using the Apoptosis Detection Kit (S7101, Sigma-Aldrich, MO, USA) following the manufacturer’s instructions. The fluorescence microscope was used to visualize the stained tissue cells.

### Statistical analysis

Statistical analysis, Student’s *t* test of one-way ANOVA, was conducted with SPSS (version 17.0) statistical software (SPSS Inc., IL, USA), and data visualization was performed with R and GraphPad Prism v8.4 (GraphPad Software Inc., CA, USA). All data are presented as means ± SD. Differences were significant when *P* < 0.05.

## Results

### Dietary restriction of methionine/cystine suppressed tumor progression in vivo

**Fig. 1:**
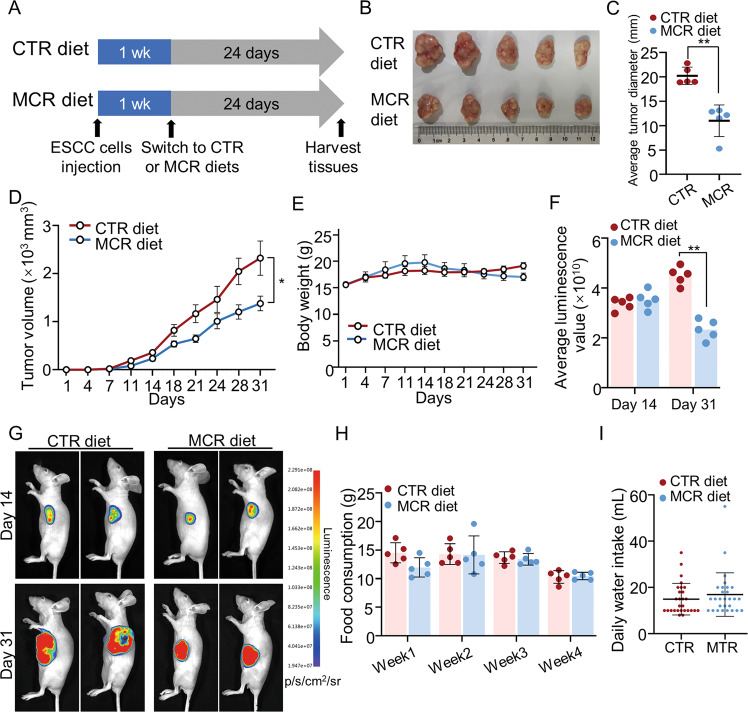
Dietary restriction of methionine/cystine suppressed tumor progression in vivo. **A** Experimental flow chart of the mice subcutaneous transplanted tumor model receiving dietary treatment intervention. A total of 10 mice randomly divided into two groups: methionine/cystine restriction (MCR) group (*n* = 5) and the control diet (CTR) group (*n* = 5). **B**–**D** After 31 days, tumors were harvested and general data on tumor were collected. The tumor images (**B**), tumor average diameter (**C**) and tumor volume (**D**) were analyzed and showed the tumor proliferation was blocked in MCR group when compared with CTR group. *n* = 5 for each group, *P < 0.05, **P < 0.01, two-tailed unpaired Student’s *t* test. **E** The Body weight of mice in MCR group and CTR group during dietary intervention. *n* = 5 for each group. **F**, **G** Luminescence scores of tumors in MCR and CTR groups at day 14 and day 31 (**F**). Representative images are shown (**G**). *n* = 5 for each group, **P < 0.01, two-tailed unpaired Student’s *t* test. **H**, **I** Weekly food consumption (**H**, *n* = 5 for each group), average daily water (**I**, *n* = 28 for each group) intake of mice in MCR group and CTR group during dietary intervention. Data are presented as mean ± SD (*n* = 3).

We performed a diet control model to confirm the effects of methionine and cystine deprivation on ESCC in a subcutaneous tumor model. ECA109 cells were subcutaneously xenografted onto BALB/c nude mice (*n* = 10) and randomly divided into two groups: one group was fed a methionine/cystine restriction (MCR) diet that contains 0.12% DL-methionine and no cystine, and the other was fed a control diet (CTR). The tumor volume, body weight, water and food intake, and other parameters were evaluated every 3–4 days during the dietary interventions (Fig. 1A).

The growth of tumors was significantly blocked in MCR group when compared with CTR group (Fig. 1B–D), whereas the body weight of mice in both groups showed no significant difference after 31 days of intervention (Fig. 1E). The average luminescence values showed the methionine/cystine deprivation slowed down the growth of the tumor (Fig. 1F, G). The weekly food consumption and daily water intake of mice in the MCR group was comparable with that in the CTR group (Fig. 1H, I). This mice model showed dietary restriction of methionine/cystine significantly blocked ESCC progression in vivo.

### Dietary restriction of methionine/cystine downregulated cellular methionine metabolism in ESCC

**Fig. 2:**
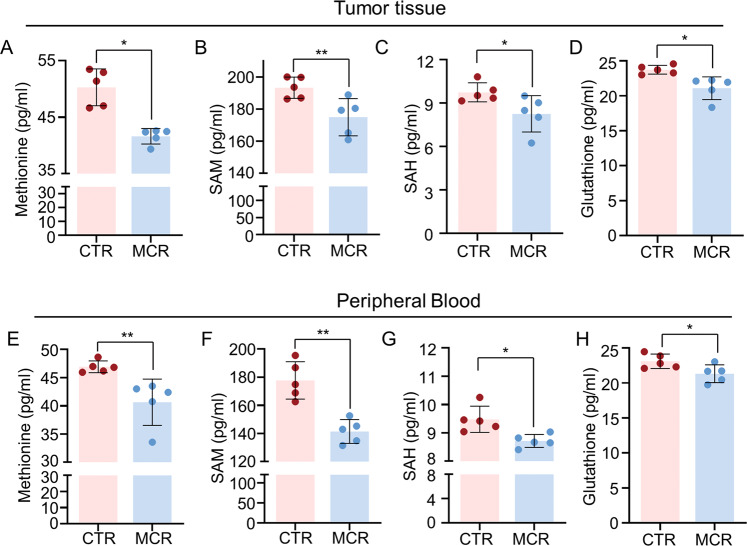
Dietary restriction of methionine/cystine downregulated cellular methionine metabolism in ESCC. **A**–**D** The content of Methionine (**A**), SAM (**B**), SAH (**C**), and Glutathione (GSH) (**D**) were detected in tumor tissues from both CTR and MCR groups when tumors tissues were harvested on day 31. **E**–**H** The content of Methionine (**E**), SAM (**F**), SAH (**G**), and Glutathione (GSH) (**H**) were detected in peripheral blood from both CTR and MCR groups when blood samples were collected on day 31st. *P < 0.05, **P < 0.01, two-tailed unpaired Student’s *t* test. *n* = 5 for each group. Data are presented as mean ± SD (*n* = 3).

To confirm the effects of MCR diet on cellular methionine metabolism, methionine, s-Adenosylmethionine (SAM), s-Adenosylhomocysteine (SAH), and glutathione (GSH) were detected in peripheral blood and tumor tissue from MCR and CTR groups. Results showed MCR diet significantly reduced the content of methionine, SAM, SAH, and GSH both in tumor tissue and peripheral blood when compared with the CTR group (Fig. 2A–H). These results confirmed MCR diet attenuated the methionine metabolism activity in ESCC, the blocked tumor progression in MCR group may relate to downregulated methionine metabolism in this mice model.

### RNA-seq and enrichment analysis of tumor tissues from MCR and CTR groups

**Fig. 3:**
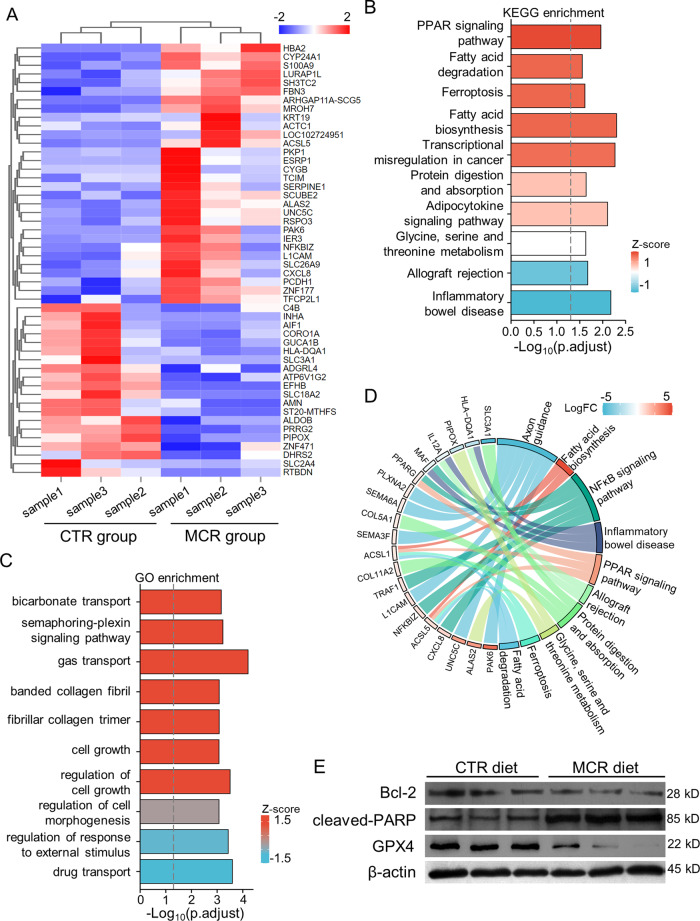
RNA-seq and enrichment analysis of tumor tissues from MCR and CTR groups. **A** The heatmap showed the top 50 differentially expressed genes. **B** KEGG enrichment analyzed on dysregulated genes. **C** The diagram showed GO term, which was based on GO annotation. **D** Chord diagrams showed a correlation between DEGs and KEGG pathway. **E** Western blot was performed to detect expression of Bcl-2, cleaved PARP and GPX4 in tumor tissues derived from the ECA109 xenograft tumor model. Tumors tissues were harvested on day 31 and tumor tissues for protein assay were kept in the –80 °C refrigerator, the experiments were performed within a week to ensure the reliable quality of tumor tissues. Data are presented as mean ± SD (*n* = 3).

RNA-seq analysis was performed on tumor tissues from MCR and CTR groups, and three randomly chosen samples were tested in each group. There was little difference in overall gene expression between each sample, no significant overexpression or underexpression samples exist (Supplementary Fig. S1A). There were 16,793 genes that were dysregulated between the two groups. Within these genes, 55 genes were significantly upregulated (*P* < 0.05; Log2FC > 0.585), 30 genes were downregulated (*P* < 0.05, Log2FC < –0.585) (Supplementary Fig. S1B). A list of all differently expressed genes (DEGs; *P* < 0.05, |Log2FC | >0.585) is shown in Supplementary Table S1 (Additional file 1). Figure 3A shows the heatmap of the top 50 DEGs.

The KEGG and GO enrichment analysis showed DEGs involved in multiple pathways and activities. The changes caused by altered methionine metabolism may lead to abnormalities in other metabolic activities, such as fatty acid metabolism and glycine, serine, and theonine metabolism. Notably, the ferroptosis and transcriptional misregulation in cancer were also induced by downregulated methionine metabolism according to the *Z*-score (Fig. 3B). The methionine/cystine deprivation also regulated cell growth and the synthesis of collagen fibril (Fig. 3C). These results showed the blocked progress of tumor in mice model caused by MCR diet may be regulated by various biological processes in cells. Because GSH was an important product of methionine metabolism and acted as an inhibitor of ferroptosis, MCR was most likely to regulated ESCC progress via cellular ferroptosis.

In-depth analysis of the correlation between the DEGs and the results of the enrichment analysis was performed. The circle plots showed the relationship between the dysregulated activities and pathways and the DEGs. In GO term, the synthesis of collagen fibers, the activated fatty acid metabolism, the negative regulation of cell migration and motility and the cell motility were correlated with MCR (Supplementary Fig. S1C). Further analysis on KEGG enrichment results showed the upregulation of ACSL1/5 regulated the fatty acid biosynthesis and degradation pathway and ferroptosis pathway. The NFκB signaling pathway was also regulated by MCR (Fig. 3D).

A network diagram was drawn with these pathways, we found these pathways were divided into two clusters (Supplementary Fig. S1D). One of them is a metabolic pathway centered on Serotonergic synapse. The other cluster was centered on cytokine–cytokine receptor interaction and Axon guidance pathway. These results suggested methionine/cystine restriction may affect different signaling pathways to regulate cellular activities and tumor proliferation. Further investigation could combine with treating specific pathway targeting drugs to find the molecular pathway in regulating cancer cell proliferation.

To verified the results of RNA-seq analysis, the protein level of Bcl-2, cleaved PARP, and GPX4 were assayed by western blot. The expression of Bcl-2, which inhibit cellular apoptosis, was downregulated in tumor tissues from MCR group when compared with CTR group, whereas the expression level of cleaved PARP, a marker for apoptosis, was higher in MCR group than in CTR group. Moreover, methionine/cystine deprivation caused downregulated the expression of GPX4, an inhibitor of ferroptosis, in tumor tissues (Fig. 3E). Our results indicated that methionine/cystine deprivation may cause ferroptosis and induced cellular apoptosis in ESCC. One study showed in murine macrophages, virus infection caused ferroptosis in a NFκB-dependent way [37], which was quite similar to our results of RNA-seq analysis and further suggested a direction to explore the mechanism of methionine metabolism regulating the development of ESCC.

The Gene Set Enrichment Analysis (GSEA) was performed to compare biological pathways in tumor tissues from MCR group and CTR group (Table 1 and Supplementary Fig. S2). Compared to the tumor tissue of the CTR group, 21 gene sets from GO database and 4 gene sets from KEGG database were regulated by changed methionine metabolism (|NES| > 1.5, NOM *P* value < 0.05). 18 of 21 gene sets from GO database being upregulated. The MCR may affect the nervous system, cell differentiation process, epithelial-mesenchymal transitions (EMT) process, cell growth in ESCC.

**Table 1:** Significantly dysregulated gene pathways in MCR group tumor tissues in gene set enrichment analysis (GSEA).

Pathway	Genes altered *n*	NES	NOM *P* value
GO_REGULATION_OF_AXON_GUIDANCE	12	2.16765	0
GO_REGULATION_OF_CYSTEINE_TYPE_ENDOPEPTIDASE_ACTIVITY	23	2.15302	0
GO_REGULATION_OF_PEPTIDASE_ACTIVITY	34	2.086	0
GO_REGULATION_OF_CELL_MORPHOGENESIS_INVOLVED_IN_DIFFERENTIATION	37	1.90549	0
GO_SEGMENTATION	10	1.88023	0
GO_CELL_DEATH	78	1.87438	0
GO_CIRCULATORY_SYSTEM_DEVELOPMENT	90	1.78643	0
GO_REGULATION_OF_CELL_DIFFERENTIATION	144	1.66515	0.001642
GO_NEUROGENESIS	111	1.63112	0.001681
GO_REGULATION_OF_CELL_GROWTH	37	1.96798	0.00177
GO_REGULATION_OF_FAT_CELL_DIFFERENTIATION	15	2.05911	0.001805
GO_REGULATION_OF_CELL_SIZE	21	2.09465	0.001835
GO_REGULATION_OF_EXTENT_OF_CELL_GROWTH	14	2.05897	0.001873
GO_REGULATION_OF_HYDROLASE_ACTIVITY	104	1.62851	0.003356
GO_POSITIVE_REGULATION_OF_PEPTIDASE_ACTIVITY	14	2.01003	0.00349
GO_NEGATIVE_REGULATION_OF_CELL_DIFFERENTIATION	62	1.74863	0.003534
GO_NEGATIVE_REGULATION_OF_CELL_GROWTH	19	1.98756	0.00365
GO_EPITHELIUM_DEVELOPMENT	86	1.70045	0.004918
GO_PHOSPHOLIPASE_C_ACTIVATING_G_PROTEIN_COUPLED_RECEPTOR_SIGNALING_PATHWAY	13	−1.7604	0.011364
GO_SECRETION	60	−1.6249	0.015113
GO_LOCOMOTORY_BEHAVIOR	18	−1.5275	0.049041
KEGG_AXON_GUIDANCE	26	1.9428	0
KEGG_HEDGEHOG_SIGNALING_PATHWAY	9	1.76627	0.00726
KEGG_HEMATOPOIETIC_CELL_LINEAGE	8	1.58435	0.045283
KEGG_PPAR_SIGNALING_PATHWAY	7	1.52438	0.047438

### MCR affected the proliferation and apoptosis of ESCC by regulating the expression of SLC43A2

**Fig. 4:**
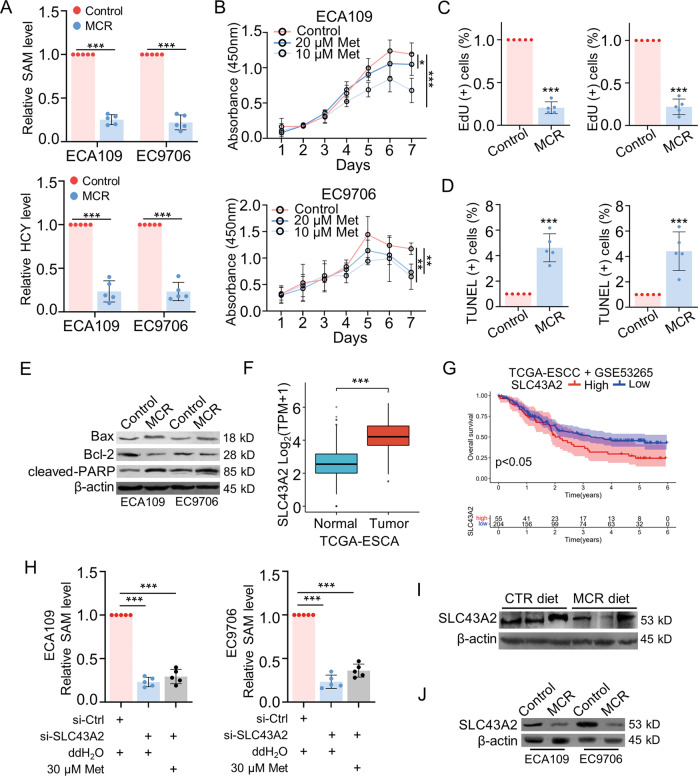
MCR affected the proliferation and apoptosis of ESCC by regulating the expression of SLC43A2. **A** The level of SAM and HCY was detected in both the control group and MCR group (cells in MCR group were cultured in the medium with 10 μM methionine and no cystine) by ELISA for 72 h. *n* = 5 for each group, ***P < 0.001, two-tailed unpaired Student’s *t* test. **B** CCK-8 assays was performed to detect the proliferation of ESCC cell lines in the control group (complete cell cultural medium), 20 μM group and 10 μM group (cells in MCR group were cultured in the medium with 10 μM or 20 μM methionine and no cystine). *n* = 5 for each group, *P < 0.05, **P < 0.01, ***P < 0.001, two-tailed unpaired Student’s *t* test, compared with the control group. **C** EdU assays were performed to detect the proliferation of ESCC in the control group and in MCR group after 72 h. The cells under proliferation were stained red under a fluorescence microscope. *n* = 5 for each group, ***P < 0.001, two-tailed unpaired Student’s *t* test, scale = 50 μm. **D** Apoptotic cells were stained green under a fluorescence microscope via TUNEL assays in control group and in MCR group after 72 h. *n* = 5 for each group, ***P < 0.001, two-tailed unpaired Student’s *t* test, scale = 50 μm. **E** Bax, Bcl-2 and cleaved-PARP expression were detected in control group and MCR group by western blot after 72 h. **F** The expression of SLC43A2 in ESCA cohort was analyzed in Tumor and Normal tissues according to TCGA database. ***P < 0.001. **G** The Kaplan–Meier analysis was performed to analyzed the correlations between overall survival rate and SLC43A2 expression in ESCC patients from TCGA database and GEO (GSE53265) database. **H** The SAM levels of ESCC cells were detected in control group, si-SLC43A2 group and si-SLC43A2 + 30 μM methionine group. All cells were cultured in complete cell culture medium after 72 h. *n* = 5 for each group, ***P < 0.001, two-tailed unpaired Student’s *t* test. **I**, **J** SLC43A2 expression in tumor tissues derived xenograft tumor model (**I**) and ESCC cells cultured in MCR condition (**J**) were detected by western blot. Data are presented as mean ± SD (*n* = 5).

The effects of methionine/cystine restriction on cell growth were further explored in vitro. The cells in the control group were incubated in complete cell culture medium. Cells in the MCR group were cultured in a methionine-restricted, cystine-deficient medium. To confirm the optimal concentration of methionine, CCK-8 assays were performed to observe the effects of different concentrations of methionine on the proliferation of ESCC cells. The results showed that the inhibitory effect on cell growth was more pronounced when cells were grown in medium with methionine content restricted to 10 μM or 20 μM, and the proliferation rate of ESCC cells did not differ significantly from the control group when the concentration of methionine starting from the 30 μM. Except for control group, culture mediums we used in other groups did not contain cystine. Therefore, cells in MCR group were cultured in the medium with 10 μM methionine and no cystine (Supplementary Fig. S3A). After 72 h incubated in methionine/cystine restriction condition, the content of SAM and homocysteine (HCY) in ESCC cells got significantly lower than that in cells cultured in complete medium (Fig. 4A). CCK-8 assays showed cell growth was significantly blocked by methionine deprivation in a dose-dependent way (Fig. 4B). The effect of methionine/cystine restriction on ESCC proliferation was also detected by EdU assay. Results showed the proliferation rate of ECA109 and EC9706 cells was decreased in MCR group (Fig. 4C and Supplementary Fig. S4A). In addition, the level of cell apoptosis was upregulated in MCR group when compared with control group as detected by TUNEL assays (Fig. 4D and Supplementary Fig. S4B). The protein levels of apoptosis-related proteins were measured by western blot. The expression of Bax and cleaved PARP was upregulated in MCR group, whereas the expression level of Bcl-2 was lower in MCR group than control group (Fig. 4E). Together, these results confirmed that methionine/cystine restriction inhibited the proliferation and induced the apoptosis of ESCC.

Our previous results indicated methionine/cystine deprivation downregulated the methionine metabolism activities in ESCC both in vivo and in vitro. Further studies focused on the regulatory mechanisms between the MCR condition and ESCC progression. As the transporter of methionine, SLC43A2 was reported as an oncogene to regulate methionine metabolism and tumor proliferation in many types of cancer [30, 38]. According to the ESCA cohort in TCGA database, the expression level of SLC43A2 was higher in tumor tissues than in normal tissues (Fig. 4F). The ESCC cohort in TCGA and GEO database (GSE53265) were combined to analyze the relationship between the SLC43A2 expression and prognosis of patients. Results showed high expression level of SLC43A2 indicated a worse overall survival rate than patients with low expression of SLC43A2 (*P* < 0.05) (Fig. 4G). These results indicated SLC43A2 may promote the ESCC progression and correlated with poor prognosis of ESCC patients. To confirm the regulatory effect of SLC43A2 on methionine metabolism, the small interfering RNA (siRNA) was used to downregulate SLC43A2 expression in ECA109 and EC9706. The SAM level was significantly downregulated by siRNA of SLC43A2 as detected by ELISA assay. Furthermore, extra methionine added in si-SLC43A2 group failed to upregulate the content of SAM in cells, which indicated SLC43A2 was necessary for transport methionine in ESCC cells (Fig. 4H). We detected the protein level of SLC43A2 in tumor tissues derived from the xenograft mouse model and in ESCC cells cultured in vitro. Results showed methionine/cystine restriction downregulated the expression of SLC43A2 when compared to the control group (Fig. 4I, J). These results indicated that MCR downregulated cellular methionine metabolism to inhibit ESCC proliferation and induced apoptosis. SLC43A2 controlled the transport of methionine in ESCC, meanwhile its expression was regulated by cellular methionine intake.

### MCR triggered a positive feedback loop between SLC43A2 and NFκB signaling pathway to regulate the proliferation and apoptosis in ESCC

**Fig. 5:**
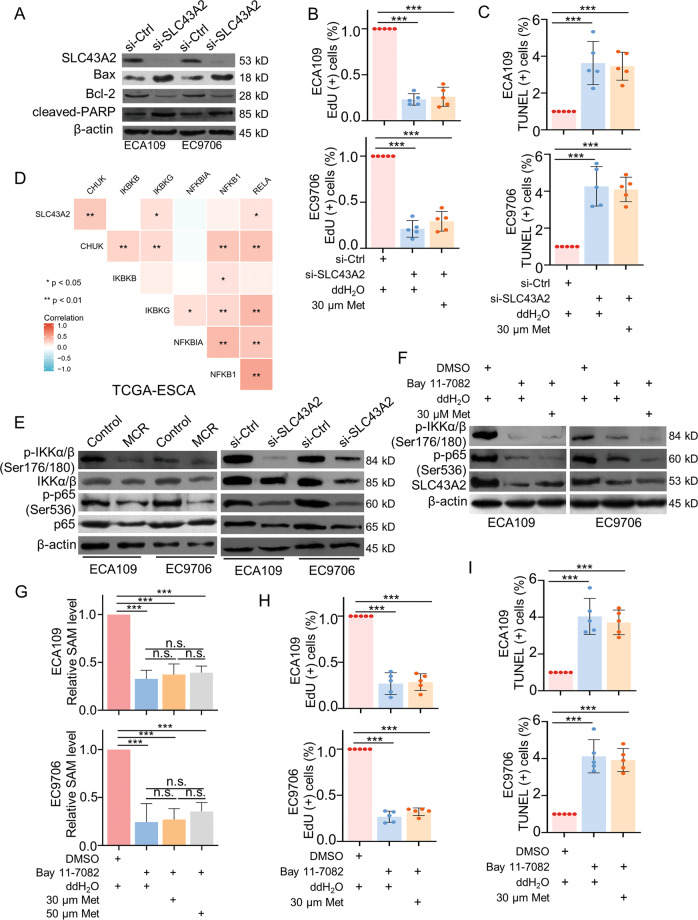
MCR triggered a positive feedback loop between SLC43A2 and NFκB signaling pathway to regulate the proliferation and apoptosis in ESCC. **A** The expression of SLC43A2, Bax, Bcl-2, and cleaved PARP were detected in ESCC cells transfected with SLC43A2 siRNA via western blot. **B**, **C** EdU assays (**B**) and TUNEL assays (**C**) were used to detected the proliferation and apoptosis of ESCC cells in (1) si-Ctrl+ddH_2_O group; (2) si-SLC43A2+ddH_2_O group; (3) si-SLC43A2 + 30 μM Met group, respectively, *n* = 5 for each group, scale = 50 μm. **D** The correlations between SLC43A2, CHUK, IKBKB, IKBKG, NFKBIA, NFKB1 and RELA in ESCA were analyzed via TCGA database, *P *<* 0.05, **P < 0.01. **E** Western blot was used to detect the expression of p-IKKα/β (Ser176/180), p-p65 (Ser536), IKKα/β and p65 in ESCC cells under MCR condition (left panel) or transfected with SLC43A2 siRNA (right panel). **F** The expression of p-IKKα/β (Ser176/180), p-p65 (Ser536), IKKα/β, p65 and SLC43A2 were detected in (1) DMSO+ddH_2_O group, (2) Bay 11-7082+ddH_2_O group; (3) Bay 11-7082 + 30 μM Met group, respectively. All cells were cultured in complete cell culture medium. **G** The content of SAM was detected respectively in (1) control+ ddH_2_O; (2) Bay 11-7082+ddH_2_O; (3) Bay 11-7082 + 30 μM Met; (4) Bay 11-7082 + 50 μM Met; *n* = 5 for each group, ***P < 0.001. **H**, **I** EdU assays (**H**) and TUNEL assays (**I**) were performed to analyzed the apoptosis and proliferation of ESCC cells, respectively, scale = 50 μm. *n* = 5 for each group, ***P < 0.001. Data are presented as mean ± SD (*n* = 5).

To verified the regulatory effects of SLC43A2 on ESCC, western blot, EdU assay, and TUNEL assay were performed in ECA109 and EC9706. Results showed the expression of BAX and cleaved PARP were upregulated by SLC43A2 siRNA, whereas Bcl-2 expression was downregulated in si-SLC43A2 group (Fig. 5A). Consistently, the proliferation was inhibited by downregulated SLC43A2 expression, extra added methionine failed to reverse this trend (Fig. 5B and Supplementary Fig. S4C). Furthermore, the proportion of apoptotic cells was increased when cells treated with SLC43A2 siRNA, extra added methionine could not decrease the apoptosis rate of cells (Fig. 5C and Supplementary Fig. S4D). These results indicated SLC43A2 could promote cell proliferation and inhibit apoptosis in ESCC.

We next explored the regulatory molecular mechanism underlying SLC43A2 and ESCC progression. Previous enrichment results suggested NFκB signaling pathway was regulated by MCR in ESCC. Our previous study confirmed activated NFκB signaling pathway significantly promoted ESCC progression and indicated a poor prognosis of ESCC patients. Through TCGA database, results showed SLC43A2 was positively correlated with CHUK, IKBKG and RELA in ESCA cohort, which encoded IKKα, IKKγ and p65, respectively (Fig. 5D). Furthermore, the expression of p-IKKα/β (Ser176/180), p-p65 (Ser536), IKKα/β, and p65 was downregulated in cells both under a MCR condition and treated with si-SLC43A2 (Fig. 5E). These results suggested methionine/cystine restriction and the silent of SLC43A2 may both block the activation of NFκB signaling pathway, which indicated that SLC43A2 could activate NFκB signaling pathway by regulating cellular methionine metabolism in ESCC. To confirm the effects of methionine metabolism on NFκB signaling pathway, methionine was added in cells that were treated with the IκB/IKK inhibitor Bay 11-7082, cells were cultured in a complete culture medium. Extra added methionine failed to rescue the expression of downregulated p-IKKα/β (Ser176/180) and p-p65 (Ser536) expression in ESCC, which may due to the expression of SLC43A2 was downregulated by Bay 11-7082. Moreover, following the methionine supplement did not rescue the SLC43A2 expression (Fig. 5F). The effects of Bay 11-7082 on cellular methionine metabolism were also detected in ESCC cells. Consistently, results showed methionine metabolism was blocked by inactivated NFκB signaling pathway, extra supplement methionine failed to rescue the side effects of Bay 11-7082 on methionine metabolism (Fig. 5G). As our previous results showed, the expression level of SLC43A2 was positively correlated with methionine content, this result mentioned that methionine metabolism-related NFκB signaling pathway activation may regulate SLC43A2 expression in ESCC. Furthermore, the methionine supplement failed to reverse the side effect of Bay 11-7082 on cell proliferation (Fig. 5H), meanwhile the apoptosis rate did not be decreased after methionine supplied (Fig. 5I and Supplementary Fig. S5A). Together, these results showed the increased methionine intake mediated by SLC43A2 activated NFκB signaling pathway, which further upregulated SLC43A2 expression and promoted ESCC progression.

### MCR induced ferroptosis by inhibiting GPX4 expression in ESCC

**Fig. 6:**
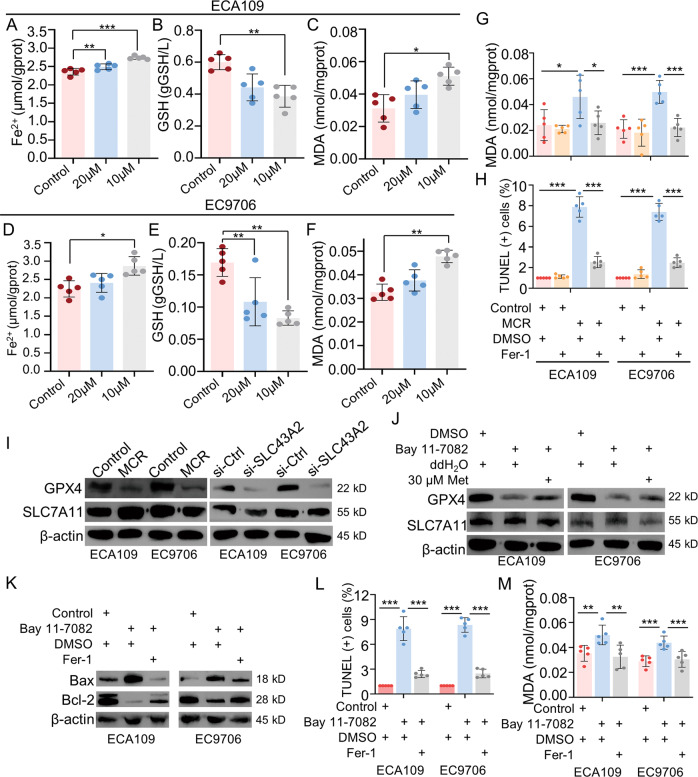
MCR-induced ferroptosis by inhibited GPX4 expression in ESCC. **A**–**F** The expressions of ferroptosis-related indexes in ECA109 and EC9706 cell lines were detected in control group, 10 μM group and 20 μM group. For 10 μM group or 20 μM group, 10 μM or 20 μM methionine was added into methionine/cystine-deficient medium, respectively. Cells were cultured for 72 h, and the content of Fe^2+^ (**A**, **D**), GSH (**B**, **E**), and MDA (**C**, **F**) in cell lysates was detected by ELISA. *n* = 5 for each group, *P < 0.05, **P < 0.01, ***P < 0.001, two-tailed unpaired Student’s t test. **G**, **H** The content of MDA (**G**) and the TUNEL-positive cells (**H**) were detected respectively in (1) control+DMSO group; (2) control+Fer-1 group; (3) MCR + DMSO group; (4) MCR+Fer-1 group. *n* = 5 for each group, *P < 0.05, ***P < 0.001, two-tailed unpaired Student’s *t* test. **I** The expression of GPX4 and SLC7A11 were detected in ESCC cells under MCR condition and transfected with SLC43A2 siRNA by western blot. **J** Western blot was used to detect the GPX4 and SLC7A11 expression in (1) DMSO+ddH_2_O group; (2) Bay 11-7082+ddH_2_O group; (3) Bay 11-7082 + 30 μM group. **K** The protein level of Bax and Bcl-2 were detected respectively in (1) control+DMSO group; (2) Bay 11-7082 + DMSO group; (3) Bay 11-7082+Fer-1 group. **L**, **M** The TUNEL-positive cells (**L**) and the content of MDA (**M**) were detected, respectively, in (1) control+DMSO group; (2) Bay 11-7082 + DMSO group; (3) Bay 11-7082+Fer-1 group. *n* = 5 for each group, *P < 0.05, ***P < 0.001, two-tailed unpaired Student’s *t* test. Data are presented as mean ± SD (*n* = 5).

Our RNA-seq-based enrichment analysis showed methionine/cystine restriction diet for ESCC xenograft mice model was a risk factor of ferroptosis. We next examined the effects of MCR on ferroptosis by in vitro experiments. ESCC cells in the control group were cultured in a complete medium. Cells in the 10 μM and 20 μM groups were cultured in medium containing 10 μM or 20 μM methionine, respectively, neither of which contained cystine. The cells were cultured for 72 h, and the contents of GSH, MDA, and Fe^2+^ were measured by ELISA assay. Results showed the MCR upregulated the production of Fe^2+^ and MDA and downregulated the content of GSH in both ECA109 (Fig. 6A–C) and EC9706 (Fig. 6D–F) in a dose-dependent way. Next, the MCR inhibitor Fer-1 was used to confirm that the apoptosis induced by MCR conditions was caused by ferroptosis (Fig. 6G). Fer-1 caused a decrease in MCR-induced cellular MDA content. When Fer-1 was added to the cells cultured under MCR conditions, the apoptosis level of the cells was successfully reduced (Fig. 6H and Supplementary Fig. S5B). The above results suggested that the elevated apoptotic levels under methionine/cystine-restricted conditions were caused by ferroptosis.

The enrichment analysis results suggested that the ferroptosis was affected by ACSL1 and ACSL5. We next examined whether the mRNA expression level of ACSL1 and ACSL5 was altered under MCR conditions. Compared with the control group, the mRNA expression levels of ACSL1 and ACSL5 in the MCR group did not show significant differences (Supplementary Fig. S3B). Furthermore, we found no difference in the expression of ACSL1 and ACSL5 between ESCA and normal tissues through the TCGA database (Supplementary Fig. S3C). To further explore the regulatory mechanism of MCR on ferroptosis, we used the TCGA database to find the correlations between SLC43A2, GPX4, the glutathione transporter SLC7A11 and key molecular from NFκB signaling pathway. Results showed the mRNA expression level of GPX4 was positively correlated with SLC43A2, IKKγ and p65, while the mRNA expression level of SLC7A11 was not correlated with SLC43A2 (Supplementary Fig. S5C). Western blot assays were further confirmed that MCR and SLC43A2 siRNA downregulated the GPX4 expression in ESCC, however, SLC7A11 expression was not affected (Fig. 6I). Furthermore, blocked NFκB signaling pathway downregulated the expression of GPX4, but not SLC7A11, which further consistent with RNA-seq results from TCGA database. Results also confirmed supplement methionine failed to rescue the GPX4 expression (Fig. 6J). The NFκB signaling pathway was next verified to affect apoptosis through the regulation of ferroptosis. Results showed Fer-1 reversed the trend of apoptosis-related protein expression induced by Bay 11-7082 (Fig. 6K). Furthermore, Fer-1 downregulated the elevated apoptosis (Fig. 6L and Supplementary Fig. S5D) and the content of MDA level induced by Bay 11-7082 (Fig. 6M). These results indicated methionine/cystine deprivation was a risk factor of cellular ferroptosis in ESCC. The SLC43A2 inhibited ferroptosis by activating NFκB signaling pathway to upregulate GPX4 in ESCC.

### The methionine metabolism-related positive feedback loop between SLC43A2 and NFκB signaling pathway promoted cell proliferation by inhibiting ferroptosis in ESCC

**Fig. 7:**
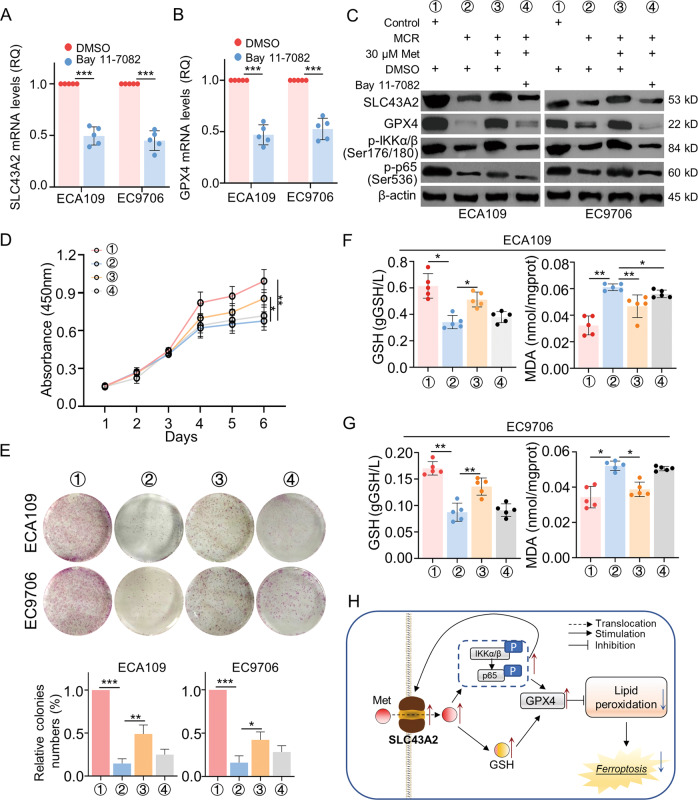
The methionine metabolism-related positive feedback loop between SLC43A2 and NFκB signaling pathway promoted cell proliferation by inhibiting ferroptosis in ESCC. **A**, **B** The qRT-PCR assay was used to detect the mRNA expression level of SLC43A2 (**A**) and GPX4 (**B**) in ESCC cells treated with Bay 11-7082. *n* = 5 for each group, ***P < 0.001, two-tailed unpaired Student’s *t* test. **C** Western blot was used to detected the expression of SLC43A2, GPX4, p-IKKα/β (Ser176/180) and p-p65 (Ser536) in ESCC cells from (1) control+DMSO group; (2) MCR + DMSO group; (3) MCR + 30 μM + DMSO group; (4) MCR + 30 μM+Bay 11-7082 group, respectively. **D** The proliferation of ESCC cell from (1) control+DMSO group; (2) MCR + DMSO group; (3) MCR + 30 μM + DMSO group; (4) MCR + 30 μM+Bay 11-7082 group was detected by CCK-8 assay. *n* = 5 for each group, *P < 0.05, **P < 0.01, two-tailed unpaired Student’s *t* test, compared with the control group. **E** The colonies formation assay was performed to detect the proliferation of ESCC cells in (1) control+DMSO group; (2) MCR + DMSO group; (3) MCR + 30 μM + DMSO group; (4) MCR + 30 μM+Bay 11-7082 group. *n* = 5 for each group, *P < 0.05, **P < 0.01, ***P < 0.001, two-tailed unpaired Student’s *t* test. **F**, **G** The content of GSH and MDA in ECA109 (**F**) and EC9706 (**G**) in certain treatments were detected by ELISA in (1) control+DMSO group; (2) MCR + DMSO group; (3) MCR + 30 μM + DMSO group; (4) MCR + 30 μM+Bay 11-7082 group. *n* = 5 for each group, *P < 0.05, **P < 0.01, two-tailed unpaired Student’s *t* test. **H** The scheme diagram showed the regulating process. SLC43A2-mediated intake of methionine activated NFκB signaling pathway, which followed the increased expression of SLC43A2 and GPX4. Meanwhile, the increased intake of methionine upregulated the content of GSH in ESCC. The feedback loop between SLC43A2 and NFκB signaling pathway diminished the ferroptosis and promote tumor progression in ESCC. Data are presented as mean ± SD (*n* = 5).

Our previous revealed a positive feedback loop between SLC43A2 and NFκB signaling pathway. MCR blocked the activation of the NFκB signaling pathway, which caused downregulated SLC43A2 expression. As a transporter of methionine, low expression of SLC43A2 impeded methionine intake and methionine metabolism activities and further inactivated the NFκB signaling pathway. The mRNA expression level of SLC43A2 and GPX4 was downregulated by Bay 11-7082 (Fig. 7A, B). In Fig. 7C, MCR downregulated the expression of SLC43A2 and GPX4, together with the phosphorylation of IKKα/β and p65. Supplement extra methionine could reverse these side effects of MCR. However, when cells treated with Bay 11-7082, methionine supplements failed to rescue the expression of SLC43A2 and GPX4 in ESCC. These results indicated the activation of the NFκB signaling pathway played an important role in regulating SLC43A2 and GPX4 expression.

The regulatory effects of this signaling pathway on cell proliferation was assayed by CCK-8 assay and colony formation assay. Results showed supplement methionine rescued the side effect of MCR on ESCC proliferation but not in cells treated with Bay 11-7082 (Fig. 7D). Consistently, when methionine added in methionine/cystine deprivation group, the cell colonies increased in both ECA109 and EC9706, however Bay 11-7082 stopped the methionine metabolism-related cell proliferation (Fig. 7E). In Fig. 7F, results showed supplement methionine rescued the content of GSH in cells under MCR condition, and downregulated the MCR-induced MDA in ECA109. When NFκB signaling pathway was blocked by Bay 11-7082, the decreased GSH and increased MDA failed to be reversed by methionine supplement. Similar results were also observed in EC9706 (Fig. 7G). These results suggested NFκB signaling pathway was important for MCR-induced ferroptosis.

Combined with the above results, MCR triggered ferroptosis and inhibited ESCC progression through a positive feedback loop between SLC43A2 and NFκB signaling pathway. The intake of methionine increased cellular glutathione and activated NFκB signaling pathway, which further caused the upregulation of SLC43A2 and GPX4. The increased SLC43A2 expression activated NFκB signaling pathway by more intake of methionine. Finally, the upregulated GPX4 blocked ferroptosis and promoted cancer progression in ESCC (Fig. 7H).

## Discussion

Here, we discovered the dietary restriction of methionine/cystine significantly blocked the ESCC progression via stimulating ferroptosis. The results showed that MCR inhibited the growth of ESCC both in vivo and in vitro. RNA-seq and enrichment analysis was performed to explore the cellular and molecular events that regulated ESCC development. Results showed ferroptosis and NFκB signaling pathway were most likely correlated with ESCC progression in MCR group. Mechanistically, MCR induced ferroptosis via a positive feedback loop between SLC43A2 and NFκB signaling pathway. Both decreased methionine intake and SLC43A2 expression diminished the phosphorylation of p65 and IKKα/β. The inactivated NFκB signaling pathway caused downregulated expression of SLC43A2 and GPX4, which further caused blocked intake of methionine and facilitated ferroptosis, respectively.

Methionine made an essential contribution to regulate intracellular redox homeostasis and ferroptosis. RNA-seq and enrichment results showed methionine/cystine restriction may affect ferroptosis, and ACSL1 and ACSL5 were related to this process. Studies showed the long‑chain acyl‑CoA synthetases (ACSLs) were deregulated in cancer, which could activated most long-chain fatty acids [39]. ACSL1 were correlated to lipid peroxidation and caused ferroptosis [37]. However, our results showed that the expression levels of ACSL1 and ACSL5 did not change after downregulation of methionine metabolism. Homocysteine synthesized from methionine produced the antioxidant glutathione (GSH) via the transsulfuration pathway, which was ultimately used to maintain the redox state of cells. The GSH was then oxidized to oxidized glutathione (GSSG) to counteract cellular damage caused by reactive oxygen species (ROS) [40]. GSH also upregulated the expression of glutathione peroxidase GPX4, a central inhibitor of ferroptosis via reducing phospholipid hydroperoxide to hydroxyphospholipid [41]. Moreover, inhibition of GSH production successfully decreased the ESCC tumor burden as assayed in a 4NQO-induced ESCC mice model [42]. These studies showed that downregulated methionine metabolism may induce ferroptosis through decreased GSH production. In nasopharyngeal carcinoma, EBV-infection-induced GPX4 combined with TAK1–TAB1/TAB3 complex activated NFκB signaling pathway [43]. Another study further confirmed p-p65 promoted the expression of LCN2 to inhibit lipid peroxidation and finally diminished the ferroptosis in hepatocellular carcinoma [44]. In our study, dietary restriction of methionine/cystine induced ferroptosis via decreased GSH and increased MDA and Fe^2+^ in ESCC. Downregulated SLC43A2 expression and NFκB signaling pathway induced ferroptosis via blocked GPX4 expression. Combined with our results, these studies suggested methionine-related NFκB signaling pathway activity may played an important role in regulating ferroptosis in ESCC. SLC43A2 has been reported to participate in methionine transport in tumor cells [30]. We indicated a positive feedback loop between SLC43A2 and NFκB signaling pathway bridging through methionine metabolism which induced ferroptosis by diminished GSH content and GPX4 expression. In hepatocellular carcinoma, PNO1 inhibition blocked SLC7A11 expression and the xCT activity followed cystine intake, which downregulated the accumulation of GSH and expression of GPX4 and induced ferroptosis [45]. As methionine could contribute to the cysteine pool via the endogenous transsulfuration pathway, our study confirmed SLC43A2 served as a significant regulator of ferroptosis via modulating the GSH content and GPX4 expression, and the obstructed NFκB signaling pathway also inhibited the expression of SLC43A2 and GPX4 in both mRNA and protein level. This regulatory model provided new therapeutic targets that may potentiate the effects of ferroptosis-based clinical antitumor therapy.

Apoptosis is a type of programmed cell death (PCD) regulated by genes and has a crucial role in the stability of normal cells in tissues, the immune and defense responses of the body, the growth and development of embryos, the development of tumors and cell damage caused by poisoning. An increasing number of studies have shown that ferroptosis and apoptosis were closely linked, that apoptosis could be converted to ferroptosis under certain conditions, and that ferroptosis promoted cellular sensitivity to apoptosis [46]. In our study, downregulated methionine metabolism induced both apoptosis and ferroptosis, and could be both downregulated by Fer-1, an inhibitor of ferroptosis, which suggested the MCR-related apoptosis was caused by upregulated ferroptosis. In addition to removing tumor cells through cell cycle arrest and apoptosis, the oncogene p53 also induced ferroptosis in tumor cells under certain conditions. In vivo and in vitro experimental studies showed that there was significant ferroptosis in addition to apoptosis in MON-P53-treated cells, which not only inhibited tumor growth, but also led to an extended lifespan in tumor-bearing mice [47]. Furthermore, we observed a significant decrease in the level of methionine metabolism and an increase in the level of apoptosis in cells after 72 h of methionine/cystine restriction, while CCK-8 assay showed that the time point at which the proliferation rate of cells in the MCR group appeared to be reduced was later than 3 days. Similar to our study, one study confirmed that U87 cells showed reduced levels of intracellular methionine metabolites after 48 h of MCR treatment, while MTT assay revealed that the proliferation level of cells was significantly reduced after 96 h [48]. In another study, the rate of tumorigenesis in vivo of CRC cells from different patient sources also showed differences in sensitivity to MCR treatment, with CRC119 showing a strong tendency to be suppressed at the beginning of the experiment, while CRC240 only showed a more pronounced trend only at the end of the experiment [23]. It was believed that this was due to differences in the dependence of methionine between different cell types or individuals [24], and the reasons for this phenomenon still need to be further explored.

Our results showed the ferroptosis was closely linked to the expression of apoptotic-related Bcl-2 family proteins. Researches showed the effect of Bcl-2 family proteins on ferroptosis may be in two ways. One was that Bcl-2 family proteins synergize against oxidative stress, for example, one study showed that co-overexpression of Bcl-2 and ΔNP63 enhanced cell resistance to oxidative stress and facilitated cell survival [49]. The other one was that Bcl-2 could directly affect the process of ferroptosis, and the specific molecular mechanism was that Bcl-2 inhibitors inactivate GPX4 by downregulating NRF2 and HMOX1, which in turn induced ferroptosis [50]. In addition, it has been found that erastin could increase Bax expression and decrease Bcl-2 expression in tumor cells [51]. The above studies suggested that Bcl-2 or other apoptotic-related proteins may be involved in ferroptosis process, and their regulatory mechanisms should be further investigated specifically in future experiments. In our study, the NFκB signaling pathway played an important role in MCR-induced ferroptosis and apoptosis. The inhibitor Bay 11-7082 induced ferroptosis and apoptosis in ESCC, and the apoptotic level was significantly reduced when cells were treated with Fer-1. Results also showed this pathway positively regulated the expression of GPX4. The above results suggested the NFκB signaling pathway inhibited cellular ferroptosis and further reduced apoptosis by upregulating GPX4. Thus, the hybrid ferroptosis/apoptosis pathway was a new strategy for cancer treatment.

Methionine played a pivotal role in cellular biological activities by providing the methyl donor S-adenosylmethionine (SAM), which participated in methylation reactions, polyamine generation, and the synthesis of cysteine and glutathione. Cells acquired methionine primarily through diet, but also regenerated methionine in small amounts through homocysteine or methylthioadenosine, reactions that intersected with the single-carbon and polyamine pathways, respectively [52]. Therefore, both methionine and cystine were restricted in the diet of animals to avoid possible resynthesis of methionine after cystine intake. Several studies have demonstrated that methionine and cystine were important for maintaining tumor growth. One study showed dietary restriction of methionine and cystine inhibits the giloma growth in a nude mouse model [48]. Other studies were also consistent with our results that methionine restriction inhibited tumor growth in breast cancer, colorectal cancer and soft tissue sarcoma [15, 21, 23, 53]. However, inconsistent results were also shown in Yang’s study [54]. Methionine/cystine restriction could inhibit A549 cell growth in vitro but not in vivo. The author performed single-cell RNA-seq and found the differential distribution of the cell cycle in vivo and in vitro may be responsible for the contradictory results, cells in the S and G2/M phase were more sensitive to methionine/cystine restriction. Our RNA-seq results did not enrich any cell cycle-related biological process, whereas results showed tumor cell growth may affect by ferroptosis. Consistently, Liu et al. reported that dual deprivation of methionine and cystine increases cellular ROS levels, decreases GSH levels, and ultimately inhibits glioma cell proliferation [48]. We found that MCR downregulated the content of GSH and the expression of GPX4 in ESCC. This provided us a new insight into how dietary methionine/cystine restriction regulated cancer progression.

Studies based on animal models have demonstrated that restricting methionine intake in the diet could extend lifespan, reduce body fat, and improve insulin sensitivity by affecting various signaling pathways [22, 55–58]. Recent studies have also shown that restricting methionine intake as a dietary intervention could induce metabolic changes that in turn improve cancer treatment outcomes [23]. However, studies had found this means of nutritional regulation exerts different effects on different human cancer cells [24, 59]. In addition to our research, another study also showed the growth rate of ESCC cells was the lowest in methionine-free media than the control group, methionine-free diet inhibit tumor growth in vivo [60]. Moreover, the large cohort studies suggested high dietary methionine intake increased esophageal cancer risk [61, 62]. These results indicated dietary methionine restriction showed great potential in improving the treatment outcome of esophageal cancer. However, few studies focused the underlying molecular mechanisms that how methionine metabolism modulated the ESCC progression. In this study, we noticed NFκB signaling pathway was regulated by methionine/cystine restriction. Both the dietary restriction and silent of SLC43A2 could diminish the phosphorylation of IKKα/β and p65, which suggested that downregulated methionine metabolism was correlated with the activation of NFκB signaling pathway. As a classical inflammation-related signaling pathway, one study indicated methionine metabolism-related NFκB signaling pathway could protect cells from inflammation induced by oxidative stress as assayed in blood and liver tissues from rats with diet supplemented with methionine [63]. The reactions to methionine may depend on tissue types as the normal cells could keep growing without methionine, but cancer cells generally couldn’t survive under the same culture conditions [64]. Considering the various roles of methionine in regulating cancer or normal tissues, more in-depth mechanism explorations were required before this dietary restriction used for clinical treatment.

In our study, the RNA-seq and enrichment results showed that altered methionine metabolism could affect the metabolism of other substances. Abnormalities in the folate cycle pathway [65] or increased demand for methionine-dependent pathways could also increase cellular methionine dependence. Alternatively, the cystine transporter xCT could be downregulated by activated PI3K in cancers, which results in enhanced cysteine synthesis via the transsulfuration pathway and a relatively reduced methionine production [29]. In our study, sequencing results showed SLC3A1, which mediated cystine transport, was significantly downregulated in MCR group. In breast cancer, upregualted SLC3A1 reduced ROS content and activated Akt signaling pathway, which resulted in promoted tumor growth [66]. Furthermore, SLC2A4, which encoded GLUT4, was also downregulated in MCR group. GLUT4 is the glucose transporter which located on cell membrane. Garrido et al. proved downregulated GLUT4 could block the proliferation of breast cancer and induce metabolic reprogramming in cancer cell [67]. Our enrichment results also showed the fatty acid degradation and biosynthesis was regulated by methionine/cystine intake. It has been demonstrated that by regulating the level of fatty acid metabolism in esophageal cancer cells, it could lead to changes in inflammatory cell function in the tumor immune microenvironment and further affect the development and progression of esophageal cancer [68]. ACSL5 and ALAS2 were downregulated in MCR group, they were all involved in fatty acid metabolism and played different roles in cancer. Downregulation of ACSL5 could be an independent prognostic factor for early recurrence of colorectal cancer [69], meanwhile dysregulated ALAS2 expression was related to the risk of acute myeloid leukemia [70]. Together, these results indicated methionine/cystine restriction may cause metabolic reprogramming in ESCC, the effects on other types of metabolism should be further studied, which would provide more theoretical basis for nutritional support therapy for ESCC.

Our study enriched the understanding of the connection among methionine/cystine metabolism and tumor progression in ESCC. The dietary restriction of methionine/cystine facilitated ferroptosis and inhibited ESCC growth both confirmed by RNA-seq analysis, animal model and in vitro assays. A positive feedback loop between SLC43A2 and NFκB signaling pathway regulated ferroptosis and tumor progression via controlling the GSH content and GPX4 expression. Moreover, MCR and silent of SLC43A2 diminished the phosphorylation of IKKα/β and p65. These results provided new insights and evidence for future research on the effects of methionine/cystine on ESCC, and provided new ideas and targets for the ESCC treatment.

## Supplementary information

Table.S1

Figure S1

Figure S2

Figure S3

Figure S4

Figure S5

Original Western blots

Reproducibility checklist

supplementary figure legneds

## Data Availability

The authors confirm the data that have been used in this work are available upon reasonable request.
