# Supplementary figures and images for: SLC43A2 and NFκB signaling pathway regulate methionine/cystine restriction-induced ferroptosis in esophageal squamous cell carcinoma via a feedback loop
Source: Cell Death Dis. 2023 Jun 3;14(6):347. doi: 10.1038/s41419-023-05860-7 (PMC10238427; doi:10.1038/s41419-023-05860-7)

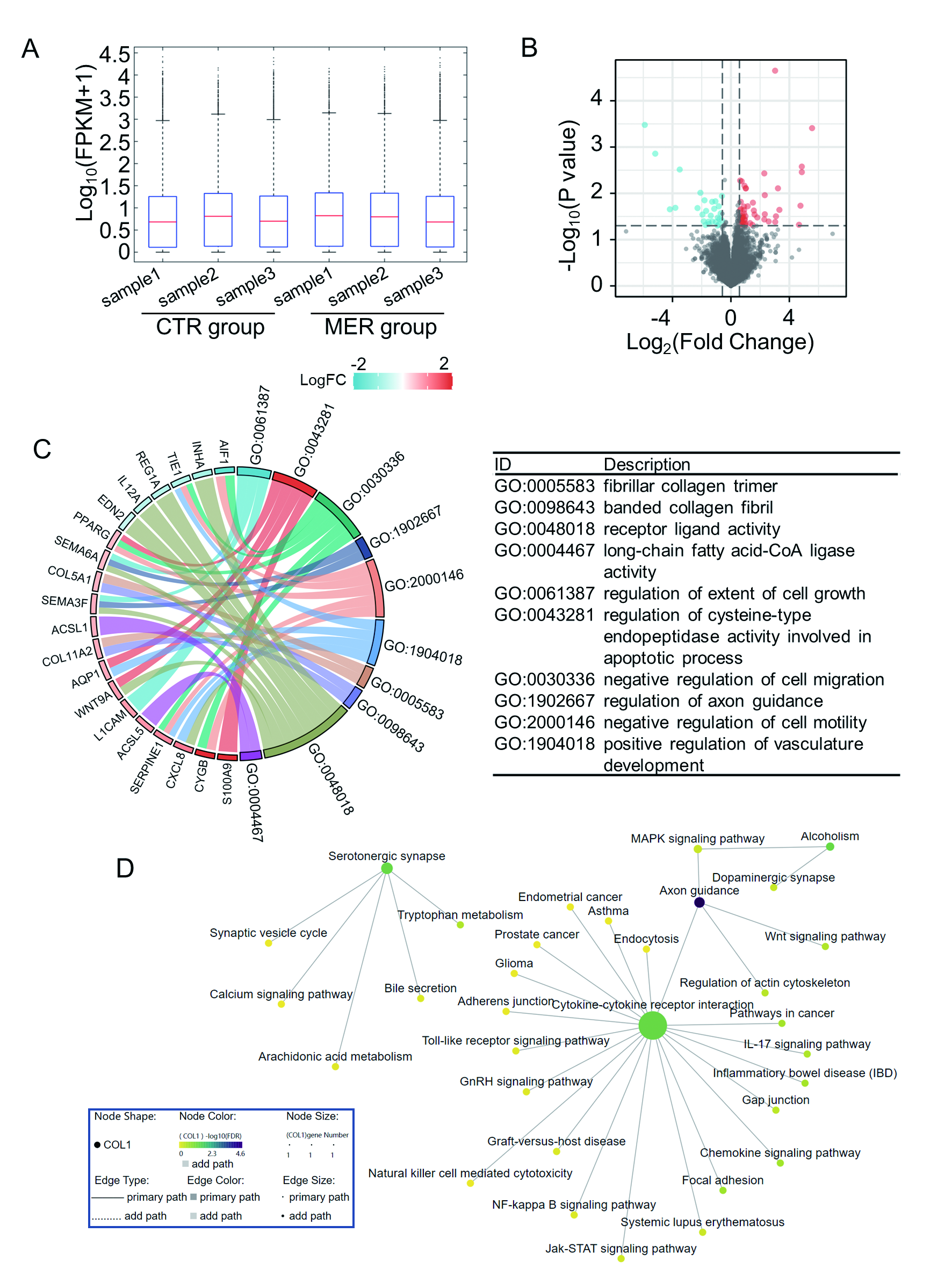

Supplement: Supplementary file 2 — Figure S1 [file 41419_2023_5860_MOESM2_ESM.tif]

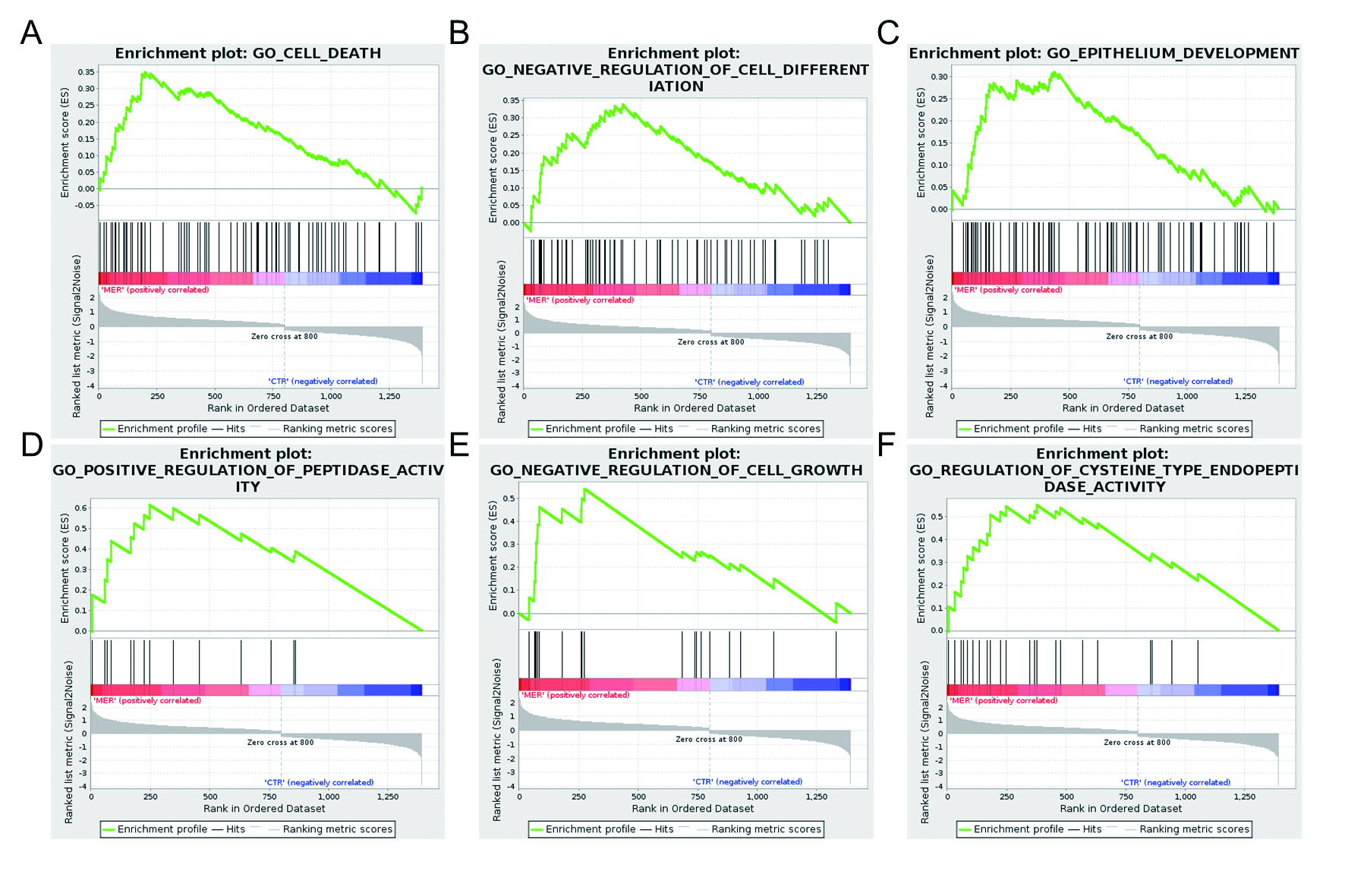

Supplement: Supplementary file 3 — Figure S2 [file 41419_2023_5860_MOESM3_ESM.tif]

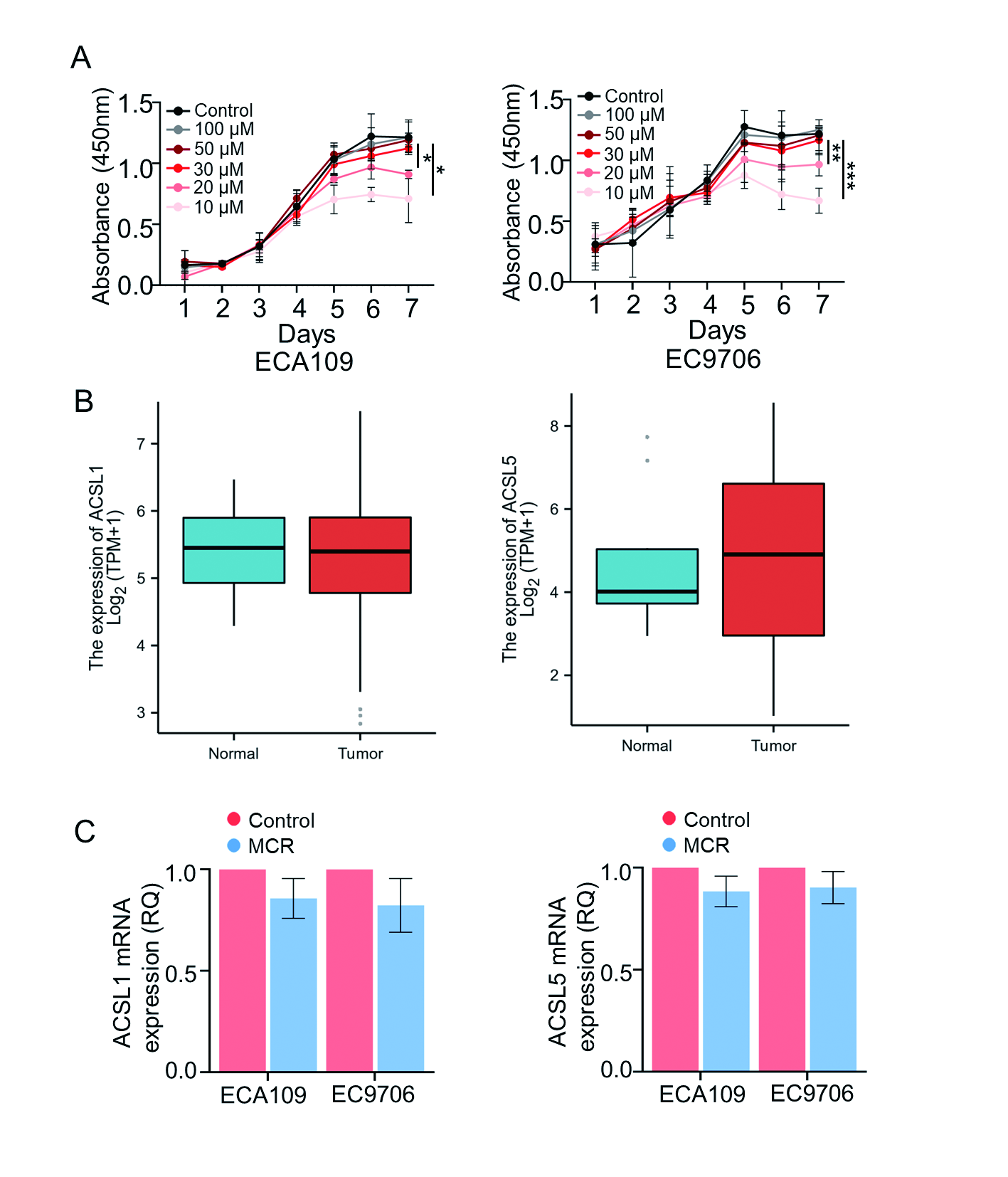

Supplement: Supplementary file 4 — Figure S3 [file 41419_2023_5860_MOESM4_ESM.tif]

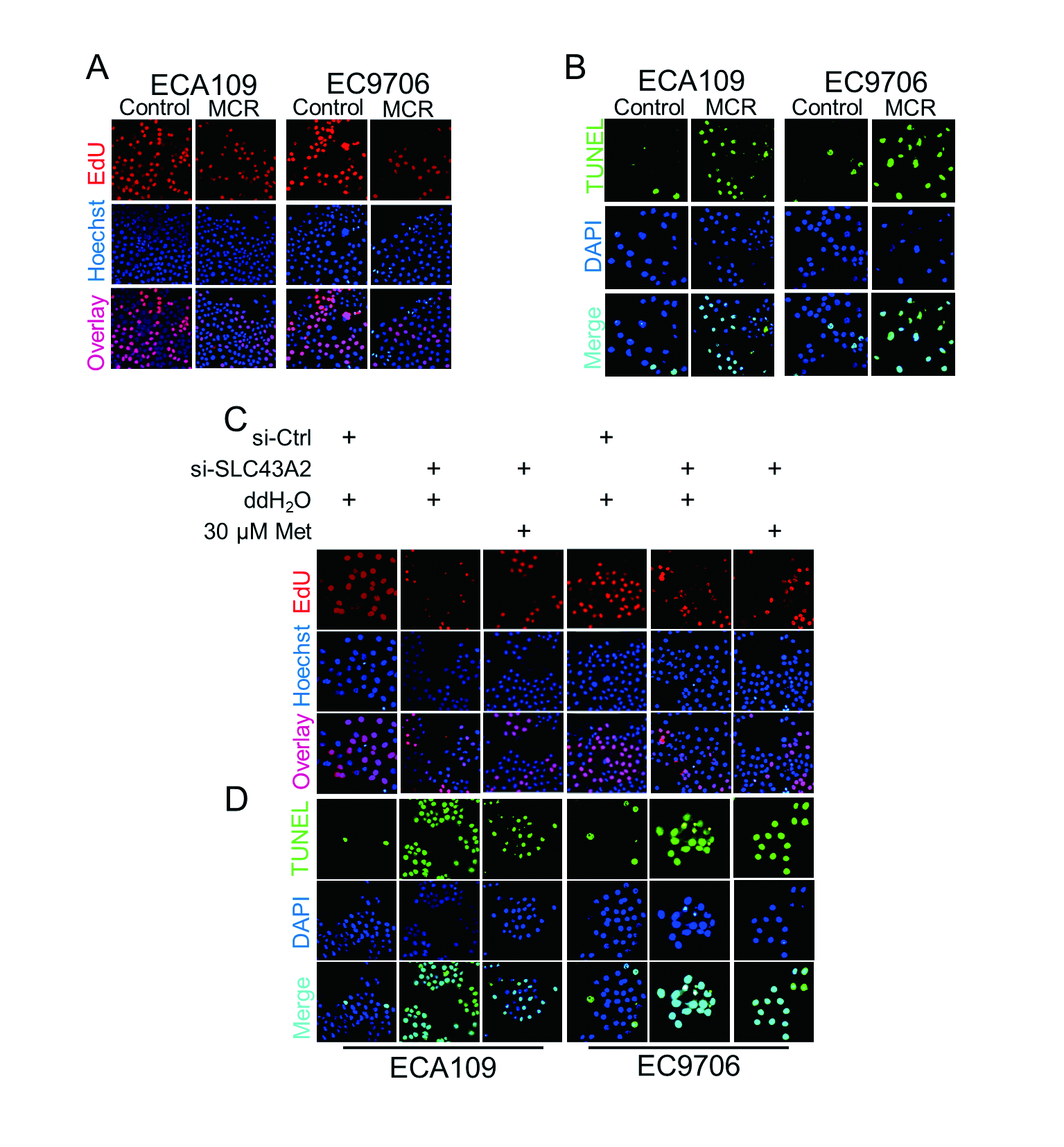

Supplement: Supplementary file 5 — Figure S4 [file 41419_2023_5860_MOESM5_ESM.tif]

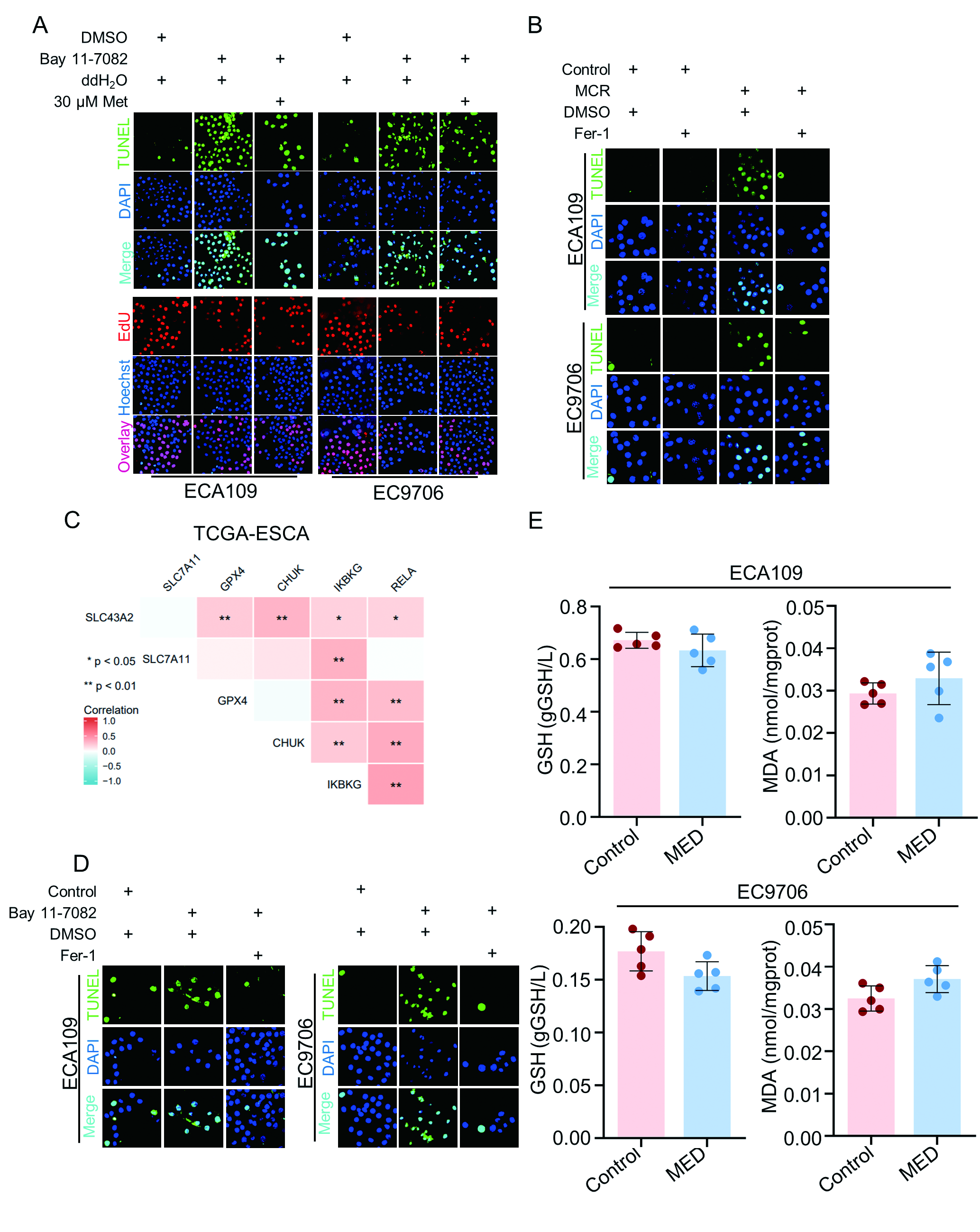

Supplement: Supplementary file 6 — Figure S5 [file 41419_2023_5860_MOESM6_ESM.tif]

Fig. 3E

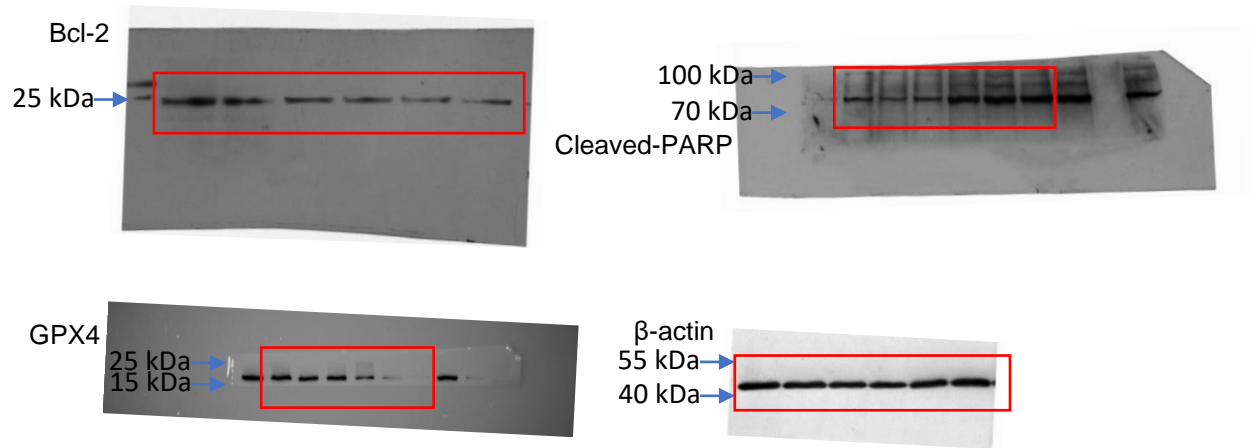

Fig. 4E

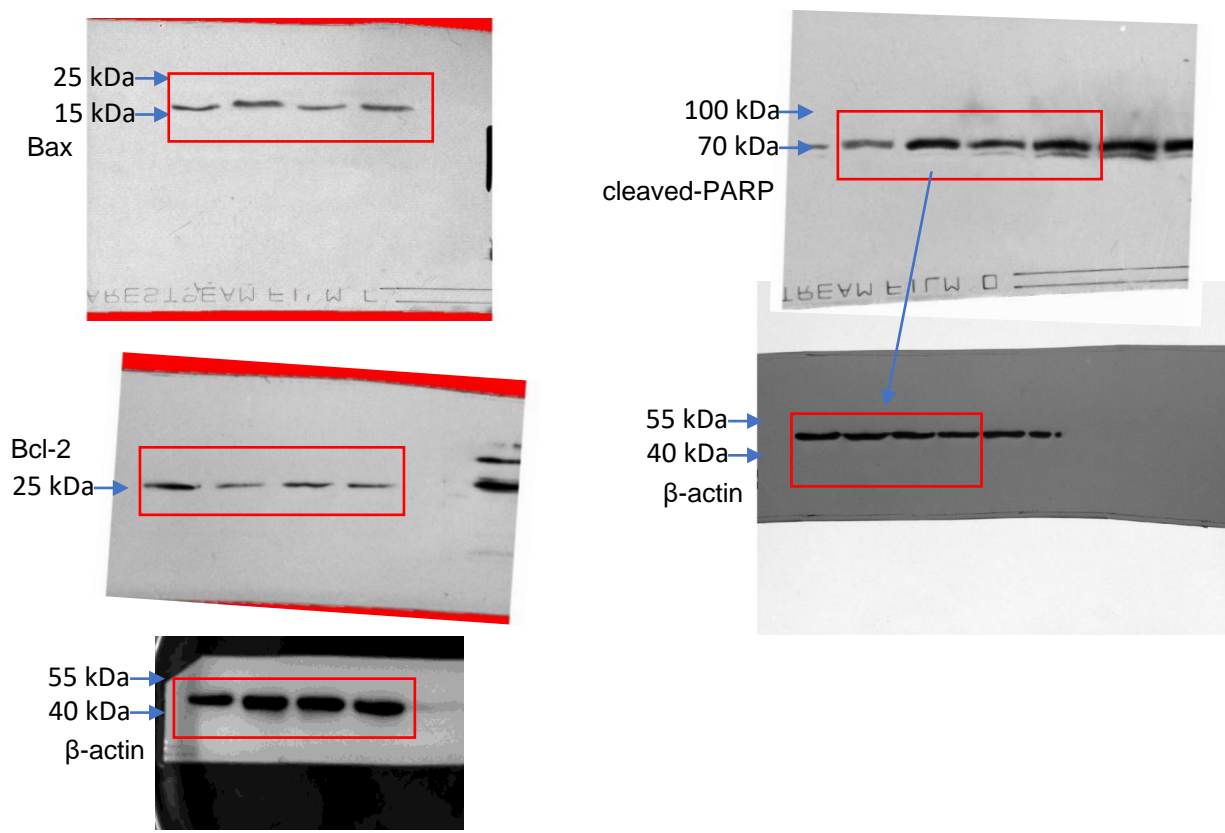

Fig. 4J

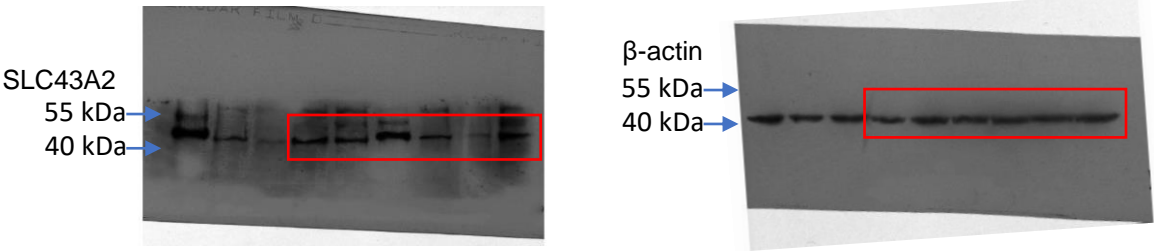

Fig. 4K

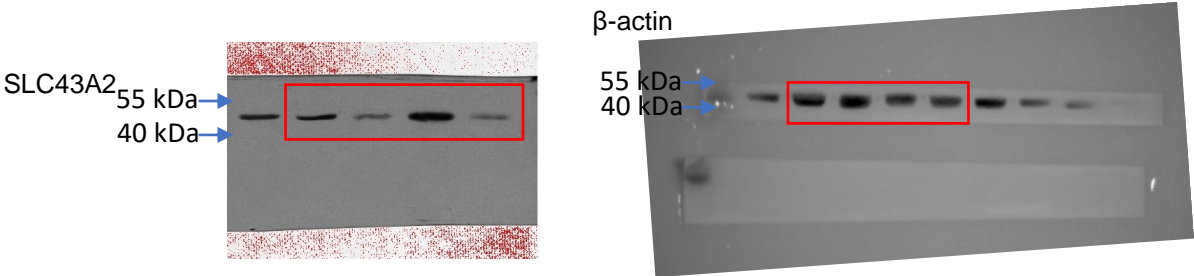

Fig. 5A

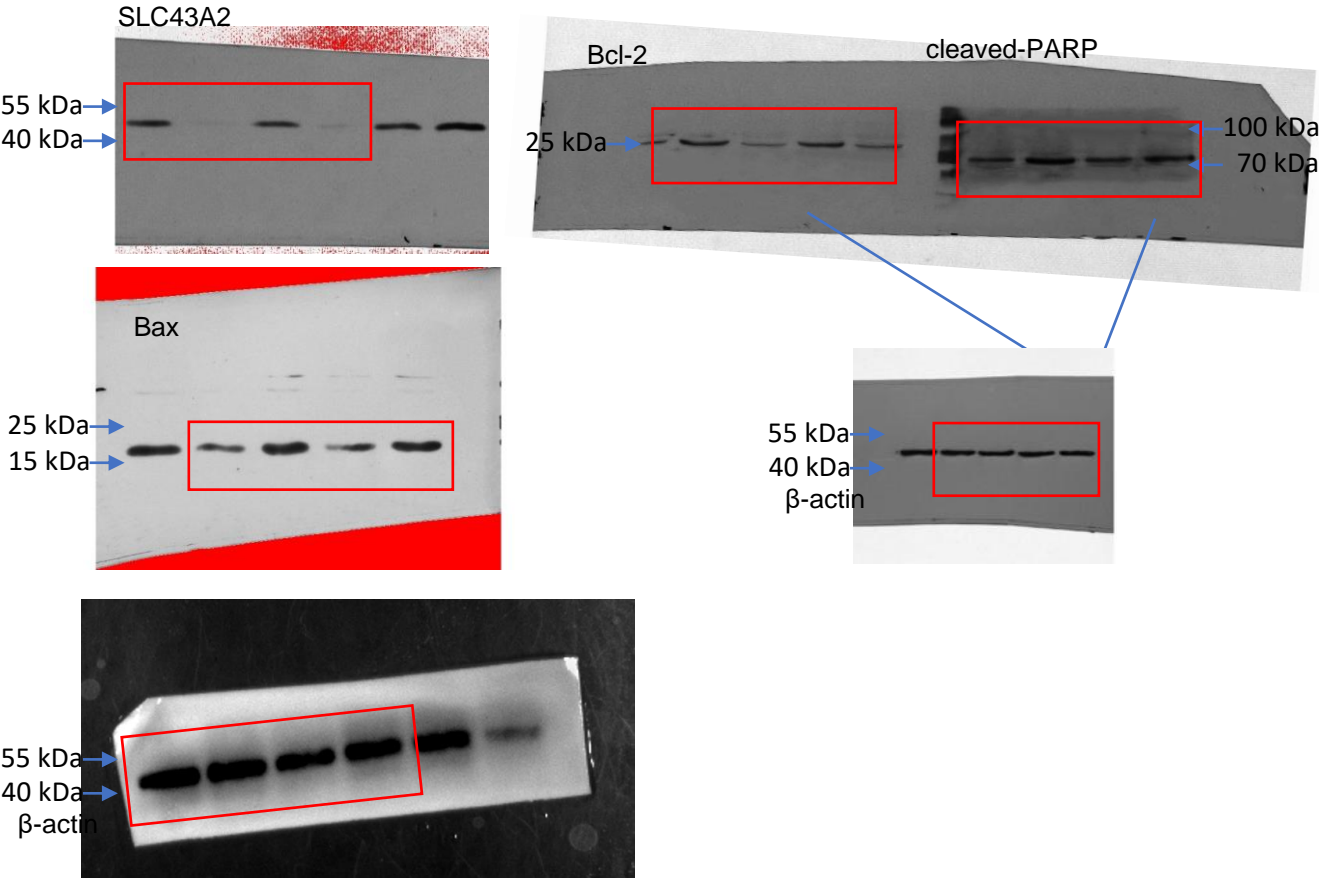

Fig. 5E

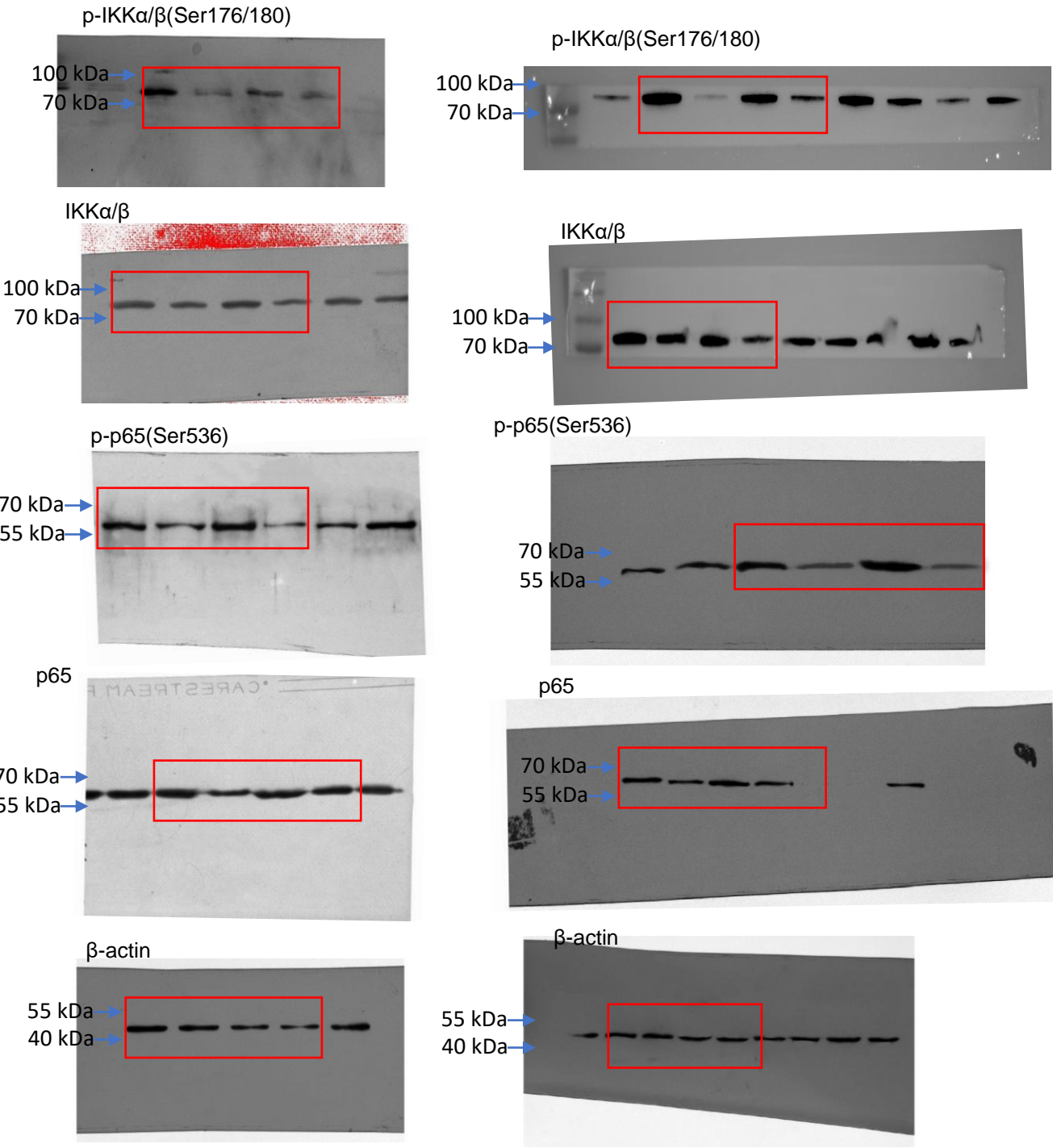

Fig. 5F

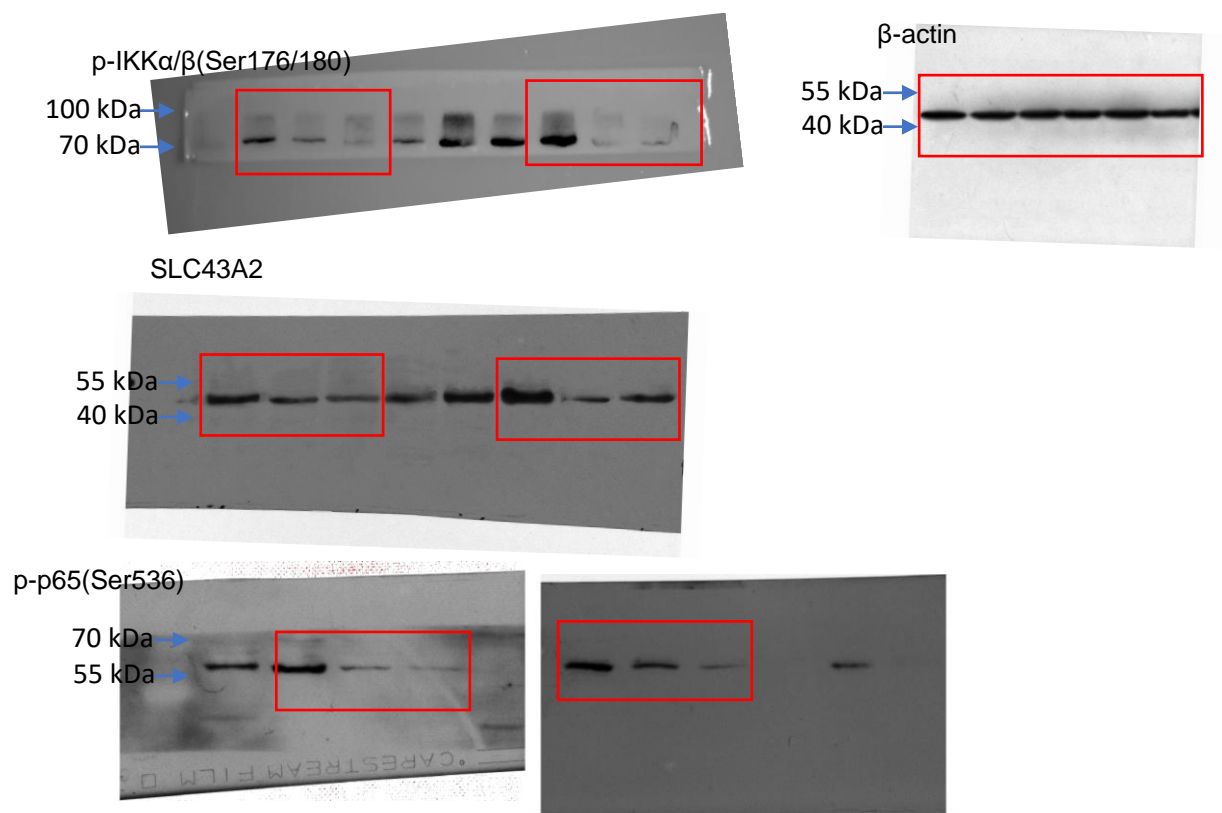

Fig. 6I

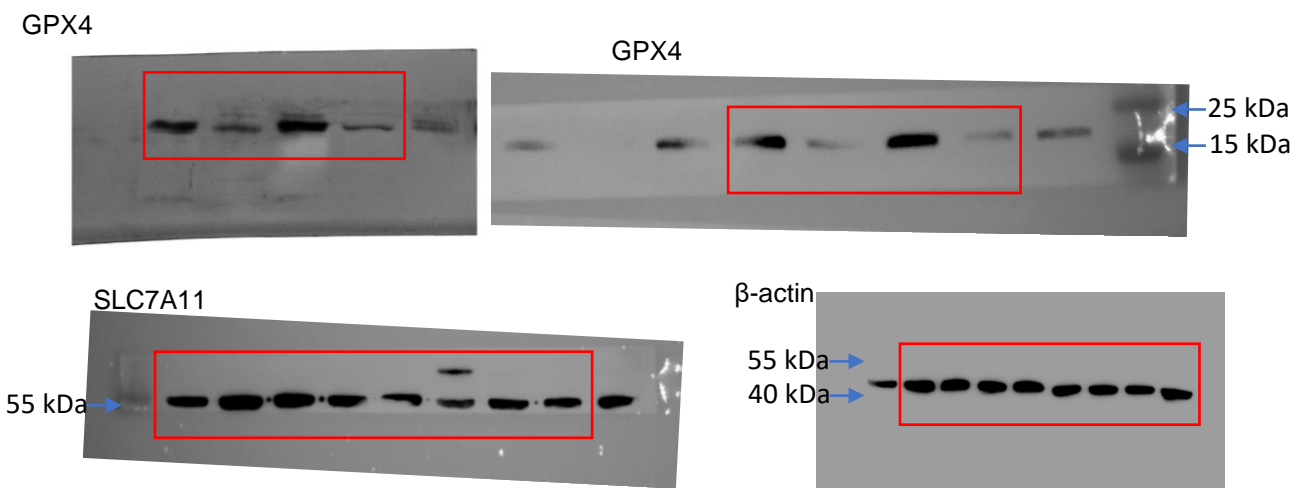

Fig. 6J

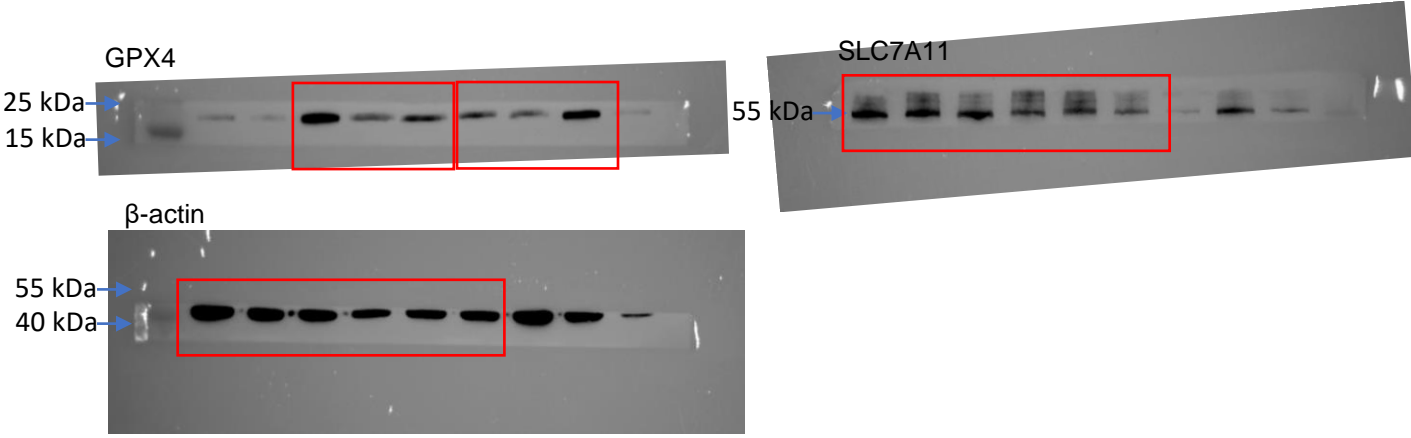

Fig. 6K

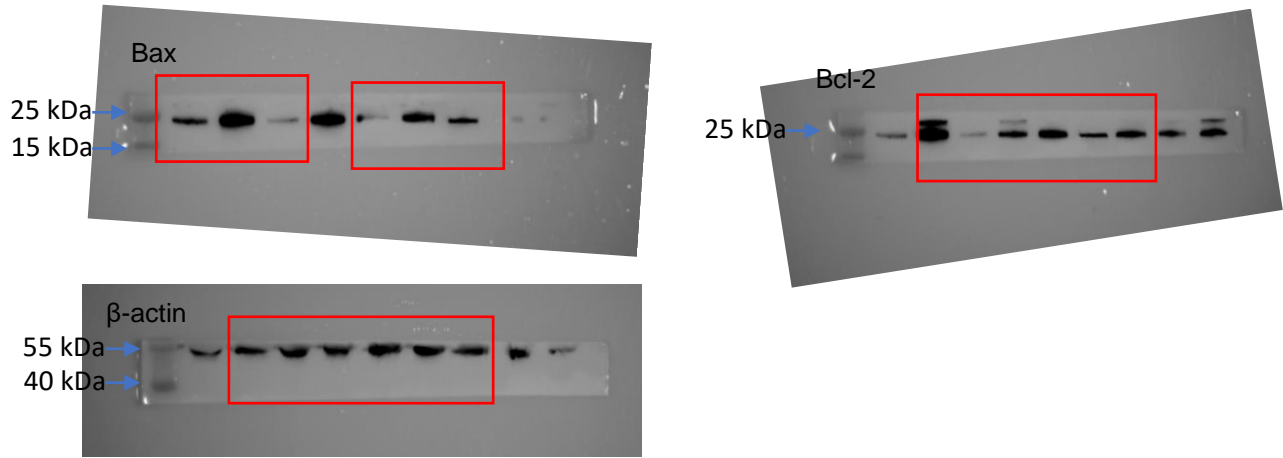

Fig. 7C

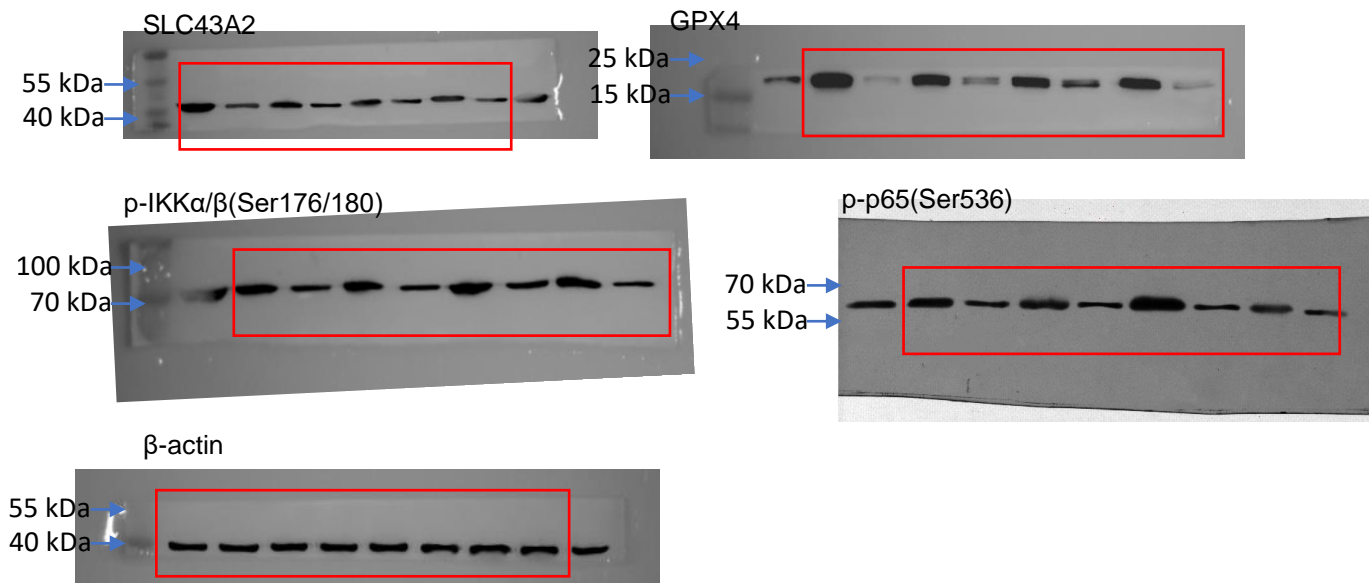

Supplement: Supplementary file 7 — Original Western blots [file 41419_2023_5860_MOESM7_ESM.pdf]
