# Supplementary material for: SLC43A2 and NFκB signaling pathway regulate methionine/cystine restriction-induced ferroptosis in esophageal squamous cell carcinoma via a feedback loop
Source: Cell Death Dis. 2023 Jun 3;14(6):347. doi: 10.1038/s41419-023-05860-7 (PMC10238427; doi:10.1038/s41419-023-05860-7)
Supplement: Supplementary file 9 — supplementary figure legneds [file 41419_2023_5860_MOESM9_ESM.docx]

**Supplementary Figure legneds**

**Fig.S1 RNA-seq and enrichment analysis of tumor tissues from MCR and CTR groups.** A. The boxplots showed analysis of expression level of protein coding genes. B. Volcanic plot showed the distribution of differentially expressed genes (P<0.05, |log2FC|>0.5850) when MCR group compared with CTR group. C. Chord diagrams showed correlation between DEGs and GO terms. D. KEGG network diagram was drawed based on KEGG enrichment analysis.

**Fig.S2 Gene set enrichment analysis (GSEA) plots of enriched pathways in tumor tissues from MCR group compared to CTR group.** A-F showed parts of enrichment results.

**Fig.S3 Effects of MCR on ESCC cells proliferation and ACSL1/5 mRNA expression.** A. To test the effects of MCR on ESCC proliferation, cells were cultured in medium lacking cystine and containing different concentrations of methionine. The culture medium contained 10 μM, 20 μM, 30 μM, 50 μM or 100 μM methionine respectively, and no cystine contained. n=5 for each group, *p<0.05, **p<0.01, ***p<0.001, two-tailed unpaired Student’s t test, compared with control group. B. The expression of ACSL1 and ACSL5 in ESCA and normal tissues were analysised based on TCGA database. C. The mRNA expression level of ACSL1 and ACSL5 were detected by qRT-PCR in control group and MCR group. n=5 for each group, two-tailed unpaired Student’s t test. Data are presented as mean ± SD (n=5).

**Fig.S4 MCR affected the proliferation and apoptosis of ESCC by regulating the expression of SLC43A2 and NFκB signaling pathway.** A and B. EdU assays (A) and TUNEL assays (B) were used to detected the proliferation and apoptosis of ESCC cells respectively in control group and MCR group. C and D. EdU assays (C) and TUNEL assays (D) were detected to analyzed the apoptosis and proliferation of ESCC cells respectively in (1)si-Ctrl+ddH_2_O group; (2)si-SLC43A2+ddH_2_O group; (3)si-SLC43A2+30 μm Met group. Data are presented as mean ± SD (n=5).

**Fig.S5 The methionine metablism-related positive feedback loop between SLC43A2 and NFκB signaling pathway promoted cell proliferation by inhibited ferroptosis in ESCC.** A. TUNEL and EdU assays were used to detected the apoptosis and proliferation in ESCC cells respectively in (1) control+ddH_2_O group; (2) Bay 11-7082+ddH_2_O group; (3) Bay 11-7082+30 μM Met group. B. TUNEL assays were performed to detect the apoptotic cells in (1) control+DMSO group; (2) control+Fer-1 group; (3) MCR+DMSO group; (4) MCR+Fer-1 group. C. The correlationship between SLC43A2, SLC7A11, GPX4, CHUK, IKBKG and RELA in ESCA were analyzed based on TCGA database. D. TUNEL assays were performed to detect the apoptotic cells in (1) control+DMSO group; (2) Bay 11-7082+DMSO group; (3) Bay 11-7082+Fer-1 group. E The contents of GSH and MDA were detected by ELISA in control group and MED (cells were cultured in medium lacking only methionine, methionine-deficient) group. Data are presented as mean ± SD (n=3).
